# Supplementary material for: Effects of music intervention combined with progressive muscle relaxation on anxiety, depression, stress and quality of life among women with cancer receiving chemotherapy: A pilot randomized controlled trial
Source: PLoS One. 2023 Nov 3;18(11):e0293060. doi: 10.1371/journal.pone.0293060 (PMC10624313; doi:10.1371/journal.pone.0293060)
Supplement: S1 File — (PDF) [file pone.0293060.s002.pdf]

Tensing instructions of 16 muscle groups in sequence

No.	Muscle	Image demonstration	Tensing Instruction
**1**	Forehead	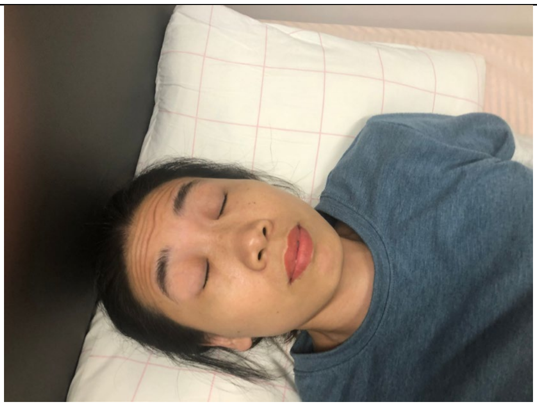	Lift eyebrows as high as possible
**2**	Eyes and nose	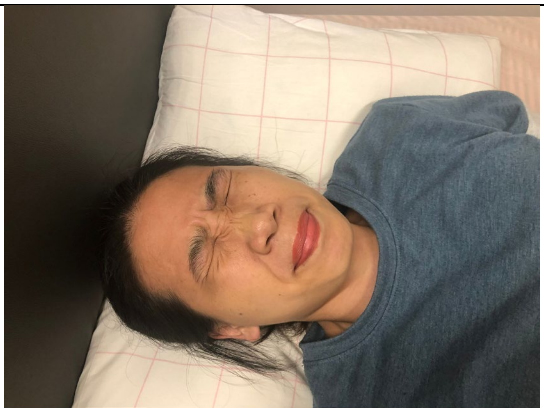	Closing eyes tightly and squinting and wrinkling nose at the same time.
**3**	Mouth and jaw	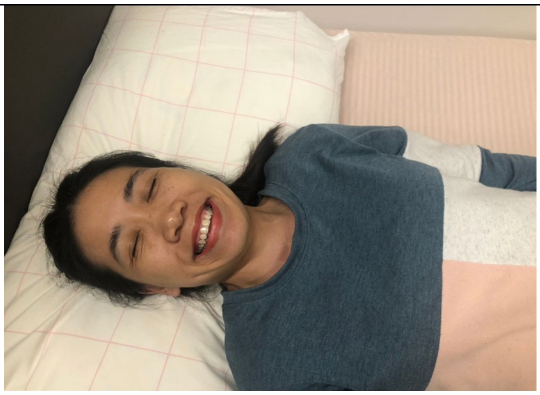	Bite hard and pull back the corners of the mouth

**4**	Neck	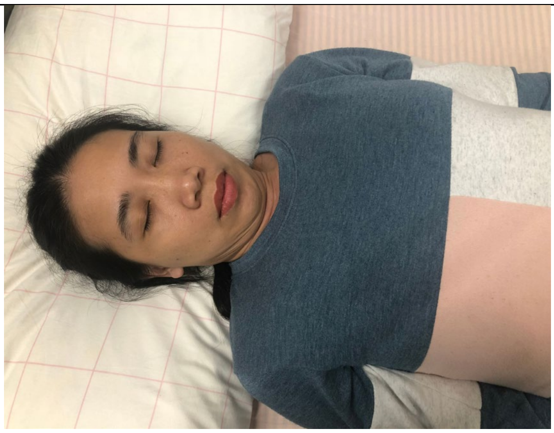	Pull the chin downward toward the chest and, at the same time, try to prevent it from actually touching the chest.
5	Dominant hand and forearm	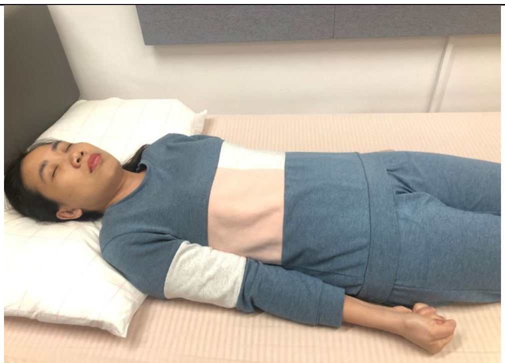	Make tight fist
6	Dominant biceps	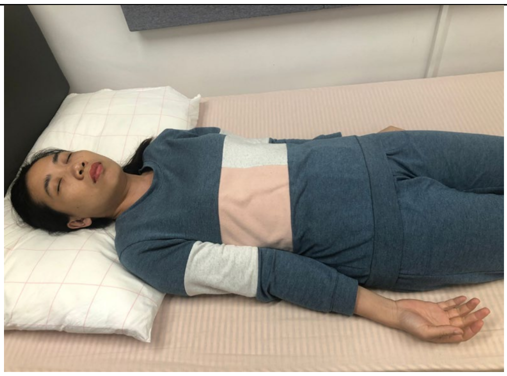	Pull and press the elbow inward toward the body
7	Non-dominant hand and forearm	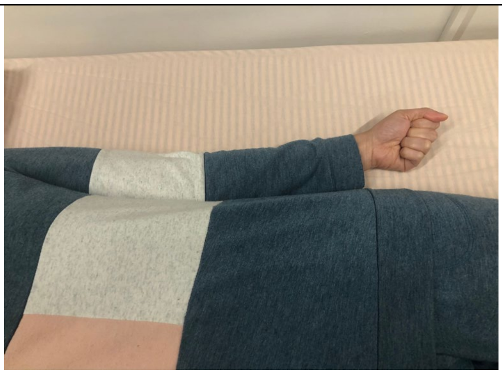	Make tight fist

8	Non-dominant biceps	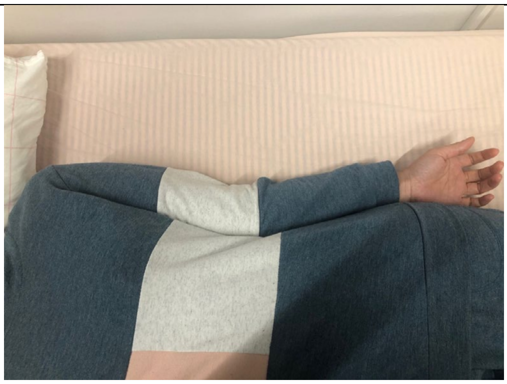	Pull and press the elbow inward toward the body
9.	Chest, shoulders, and upper back	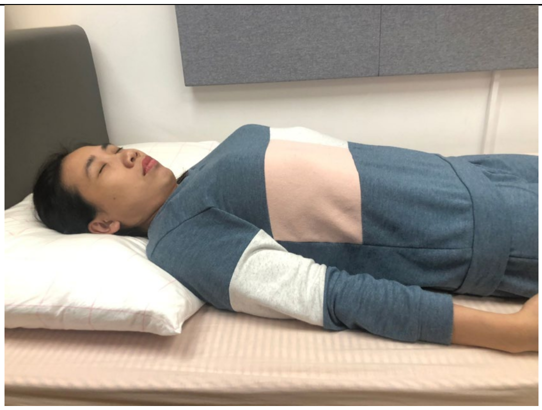	Taking a deep breath, hold it, and at the same time, pull the shoulders back and try to make the shoulder blades touch
10	Abdomen	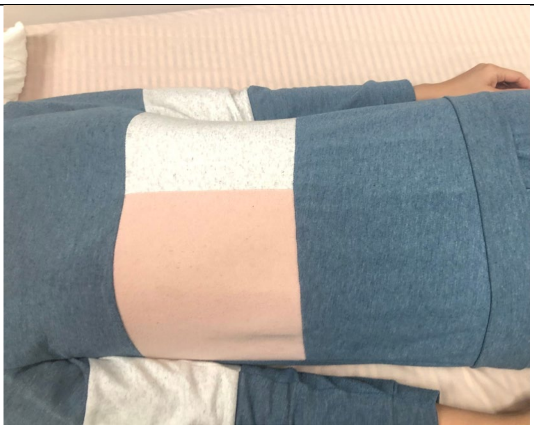	Take a deep breath and tighten the abdomen as much as you can
11.	Dominant the upper leg	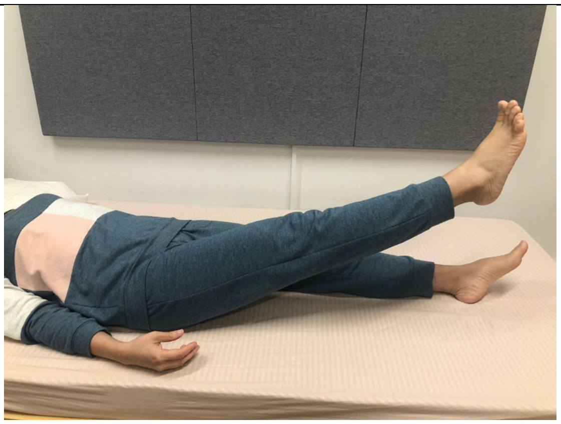	Lift the leg a little bit

12	Dominant calf	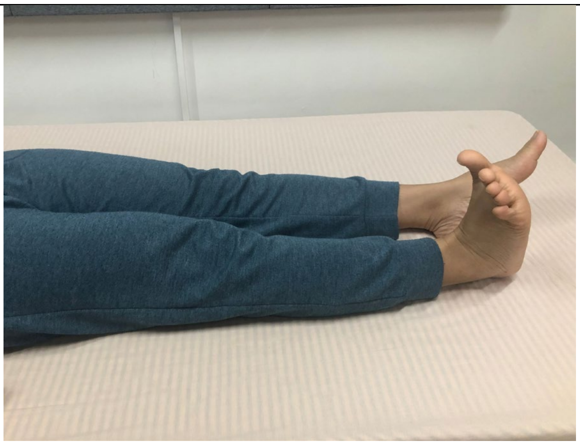	Pull the toes upward toward the head
13	Dominant foot	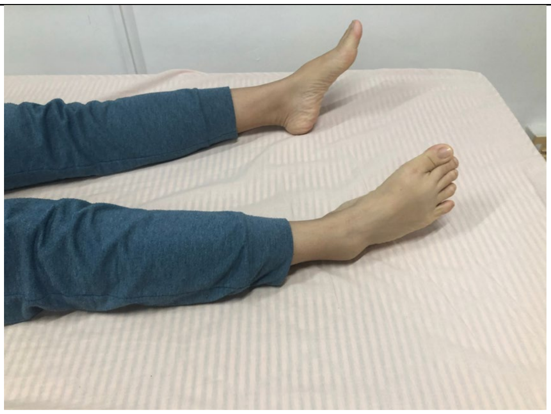	Point the toes, turn your foot inward, and at the same time, curl the toes. (not more than 5 seconds to prevent cramps)
14	Nondominant upper leg	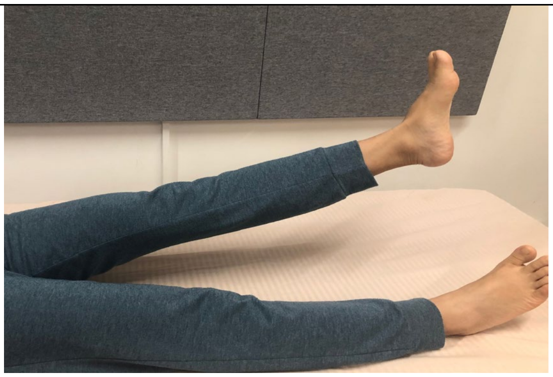	Lift the leg a little bit
15.	Non-dominant calf	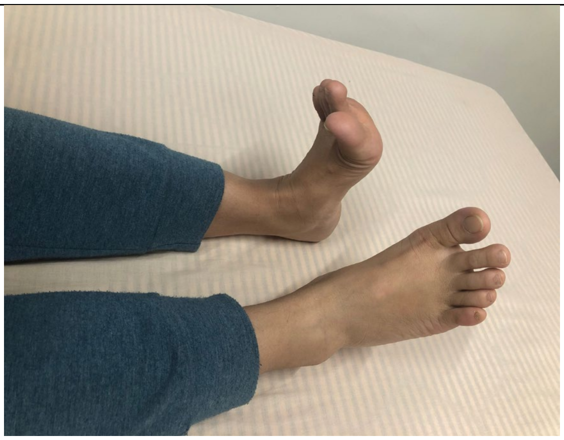	Pull the toes upward toward the head

16.	Non-dominant foot	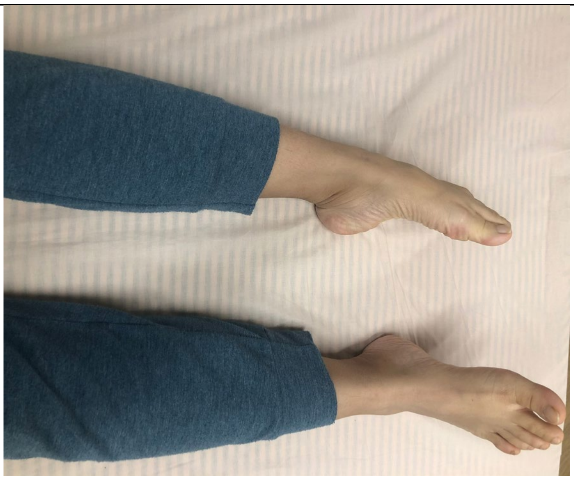	Point the toes, turn your foot inward, and at the same time, curl the toes. (not more than 5 seconds to prevent cramps)
