# Supplementary material for: Effects of music intervention combined with progressive muscle relaxation on anxiety, depression, stress and quality of life among women with cancer receiving chemotherapy: A pilot randomized controlled trial
Source: PLoS One. 2023 Nov 3;18(11):e0293060. doi: 10.1371/journal.pone.0293060 (PMC10624313; doi:10.1371/journal.pone.0293060)
Supplement: S2 File — (DOCX) [file pone.0293060.s003.docx]

**The Chinese University of Hong Kong**

**Faculty of Medicine**

**The Nethersole School of Nursing**

**Doctor of Philosophy in Nursing Program**

**2020-2021**

**Study protocol**

**Effects of music intervention combined with progressive muscle relaxation on anxiety, depression, stress and quality of life in breast and gynaecological cancer patients receiving chemotherapy: A randomized controlled trial**

**PhD student: Khanh Thi Nguyen**

**Student ID: 1155150008**

**Supervisor: Prof. CHAN YIP Wing Han, Carmen**

**November 2021**

Table of Contents

[Introduction 5](#_Toc88727464)

[Literature review 8](#_Toc88727465)

[1. Effectiveness of MI on anxiety, depression and QoL 8](#_Toc88727466)

[2. Progressive muscle relaxation on anxiety, depression and QoL 8](#_Toc88727467)

[3. Effect of MI combined with PMR on anxiety, depression and QoL in cancer patients 9](#_Toc88727468)

[Qualitative study 12](#_Toc88727469)

[Intervention development 13](#_Toc88727470)

[1. Theory-guided study development 13](#_Toc88727471)

[2. Contents and dose of MCP intervention 15](#_Toc88727472)

[3. PMR and music preparation 15](#_Toc88727473)

[4. MCP Tranining program 16](#_Toc88727474)

[5. Content validation of MCP protocol 16](#_Toc88727475)

[Randomized controlled trial 18](#_Toc88727476)

[Pilot study 18](#_Toc88727477)

[1. Aim and objectives 18](#_Toc88727478)

[2. Sample size estimation 18](#_Toc88727479)

[Main study 19](#_Toc88727480)

[1. Aim and objectives 19](#_Toc88727481)

[2. Study design 19](#_Toc88727482)

[3. Participants 20](#_Toc88727483)

[4. Settings 21](#_Toc88727484)

[5. Study sample 21](#_Toc88727485)

[6. Randomization and allocation concealment 21](#_Toc88727486)

[7. Blinding 22](#_Toc88727487)

[8. Outcome measures and instruments 22](#_Toc88727488)

[9. Procedure 23](#_Toc88727489)

[10. Intervention fidelity 27](#_Toc88727490)

[11. Data analysis 27](#_Toc88727491)

[12. Process evaluation 28](#_Toc88727492)

[4. Ethics 29](#_Toc88727493)

[5. Study significance 30](#_Toc88727494)

[Study plan 31](#_Toc88727495)

**List of abbreviations**

| BCG | Breast or gynaecological cancer |
| --- | --- |
| MI | Music intervention |
| PMR | Progressive muscle relaxation |
| SCT | Stress and Coping Theory |
| QoL | Quality of life |
| MRC | Medical Research Council |
| MCP | Music intervention combined with progressive muscle relaxation |

# Introduction

In Vietnam, breast cancer has the highest age-standardized incident rate (34.2/ 100.000) among women cancer and is the third leading cause of cancer death in both sexes (13.8/100.000) (Ferlay J et al., 2020). Cervical cancer has been the second most common cancer in females from 15 to 44 years old (Nguyen et al., 2018) in Vietnam. According to Van Minh et al. (2017), cervical cancer was the leading cause of cancer death among women in South Vietnam and the second leading cause of cancer death in North Vietnam. Psychological issues for cancer patients during chemotherapy treatment are receiving much attention recently. Anxiety and depression are the most common psychological issues in breast and gynaecological cancer (BGC) patients, and the problem increases during chemotherapy. The percentage of women diagnosed with BGC that reported moderate to severe levels of depression was 24% (Ell et al., 2005). It is reported that BGC patients had higher anxiety and depression scores during chemotherapy and the issues were maintained over time (Schwarz et al., 2008). Around 40% of BGC patients receiving chemotherapy experienced moderate to severe depression (Ramezani, 2001; Shakeri et al., 2009). Depression caused a low quality of life (QoL) in BGC cancer patients (Shakeri et al., 2016). In Vietnam, 43.1% of cancer patients had anxiety during hospitalisation, 15.5% out of that had an anxiety disorder ( ≥ 11 HADS score) (Truong et al., 2019). Around half of Vietnamese cancer patients experienced anxiety and depression during chemotherapy (Hoang et al., 2020).

To manage these psychological issues, pharmacological and non-pharmacological treatments have been applied. Although pharmacological intervention showed the effect on psychological treatments, it can cause a variety of side effects (Ballenger, 2000). Moreover, using medicine should be considered drug-drug interactions, cost of treatment (Ballenger, 2000). Thus, non-pharmacological interventions have been introduced to minimize the adverse effects of cancer treatment. Among non-pharmacological interventions, music intervention (MI) and progressive muscle relaxation (PMR) are more feasible to implement than others because they are inexpensive and safe, easy to be accepted (Bulfone et al., 2009; Jasemi et al., 2016; Yilmaz & Arslan, 2015), easy to apply in Vietnamese clinical settings.

MI is considered mind-body medicine, (Tascilar et al., 2006) which can improve the patient's physical, mental, and spiritual well-being or maintain the health of cancer patients (Burrai et al., 2014). Musical reactions are recognized by the amygdala and hippocampus (Miaskowski et al., 2017). The rhythm and melody of relaxing music act on the peripheral and hippocampus system could reduce cortisol levels (Thoma et al., 2013) and improve the psychological disorder (Chen et al., 2020). MI had a beneficial effect on anxiety in people with cancer (Bradt et al., 2016). A previous study showed that music could reduce anxiety, depression, and improve the QoL for cancer patients during chemotherapy (Lima et al., 2020).

PMR is commonly used for stress management. When the body is faced with stress, the body produces a series of reactions, including the tense response of muscle groups (Garmany, 1952). The principle of PMR is based on the tension and relaxation of muscles to change the emotions of the body. Importantly, muscle is supplied by both motor and sensory nerves. Muscle contractions cause electrical impulses and are transmitted to the central nervous system (Garmany, 1952). It causes disturbances in the central nervous system and causes emotional changes.

Numerous studies evaluated the effect of MI and MPR alone. A combination of MI and PMR intervention was evaluated on breast cancer patients after radical mastectomy by K. N. Zhou et al. (2015). The results showed a significant effect of the combined intervention on anxiety and depression. Moreover, Liao et al. (2018b) proved that a combination of MI and PMR was more effective than PMR alone. However, to our best knowledge, there was no study assessing the effect of MI combined with PMR on anxiety, depression and QoL in BGC patients receiving chemotherapy. Combining music intervention (MI) and progressive muscle relaxation (PMR) may be a more effective direction for BGC patients receiving chemotherapy to manage psychological issues. This paper will provide the evidence-based protocol for evaluating the effects of MI combined with PMR. This protocol will follow the Medical Research Council (MRC) framework (Craig et al., 2013) for the development and evaluation of complex intervention that will include four phases the development, feasibility and piloting, evaluation and implementation. For the development phase, the literature review was done to identify the best evidence for intervention development. A qualitative study was conducted to understand the music perception, music preference of BGC in Vietnam. The qualitative study results help to develop the intervention that adapts Vietnamese culture. The Stress and Coping theory (Lazarus, 1984) will be used to underpin this study. Before conducting the randomized controlled trial to evaluate the effect of MI combined with PMR, a pilot study will be done to test acceptability among BGC in Vietnam.

# Literature review

1. **Effectiveness of MI on anxiety, depression and QoL**

We conducted a systematic review and meta-analysis to review the effect of MI on anxiety, depression and QoL of adult cancer patients receiving chemotherapy, and to identify the best available evidence concerning the characteristics of MI such as duration, number of sessions and frequency which could be applied on adult cancer patients receiving chemotherapy. Fourteen databases were searched from the inception date to December 2020 to identify eligible Randomized Controlled Trials (RCTs). Grey Literature was also examined.

Nine RCTs (Bro et al., 2019; Bulfone et al., 2009; Burns et al., 2008; Chen et al., 2020; Ferrer, 2007; Lima et al., 2020; Lin et al., 2011; Mondanaro et al., 2020; Tuinmann et al., 2017) were identified among which six (Bulfone et al., 2009; Chen et al., 2020; Ferrer, 2007; Lima et al., 2020; Lin et al., 2011; Tuinmann et al., 2017) were eligible for the meta-analysis. The pooled results reveal that music intervention could reduce anxiety (SMD: −0.29, 95% CI −0.50 to −0.08), and improve QoL (SMD: 0.42, 95% CI −0.02 to 0.82). However, it fails to affect depression (p=0.79). The findings of sub-group analysis suggest that the duration of a music listening session from 15 to 20 minutes, delivering immediately before chemotherapy is more effective to reduce anxiety. It is recommended to choose relaxing music with a tempo of 60 to 80 beats/min (Bro et al., 2019; Bulfone et al., 2009; Chen et al., 2020)

1. **Progressive muscle relaxation on anxiety, depression and QoL**

The second systematic review aims to review the effectiveness of progressive muscle relaxation PMR on anxiety, depression, QoL on cancer patients receiving chemotherapy. Literature was searched from 12 databases and seven RCTs (Arakawa, 1997; Dikmen & Terzioglu, 2019; Gok Metin et al., 2019; Herizchi et al., 2012; Lerman et al., 1990; Song et al., 2013; Yilmaz & Arslan, 2015) was included in this review.

Three studies (Arakawa, 1997; Song et al., 2013; Yilmaz & Arslan, 2015) showed a significant effect on anxiety. A pool effect size of those studies illustrated a large effect on anxiety (SMD: -1.05, 95% CI −1.33 to −0.76, I^2^= 29%). Arakawa (1997) recruited 60 cancer patients undergoing chemotherapy. A nurse-led PMR with deep breath was given to the patients within 72 hours of hospitalization. The dose was 25 minutes/session and twice/ day. Yilmaz and Arslan (2015) revealed a significant effect of PMR on breast cancer after 3 weeks of practising with 3 times/ weeks. However, Lerman et al. (1990) that delivered 30 minutes of PMR, once/day through three chemotherapy treatments indicated that there was no benefit of PMR on anxiety and depression. Herizchi et al. (2012) showed that although practising PMR two to three times a day could not reduce anxiety, depression and improve QoL after 1 month, it gave opposite results after three months. PMR had no significant effect on QoL on gynaecological cancers and breast cancer patients undergoing chemotherapy (Dikmen & Terzioglu, 2019; Gok Metin et al., 2019).

1. **Effect of MI combined with PMR on anxiety, depression and QoL in cancer patients**

Nurse-led MI combined with PMR (MCP) had a significant effect on anxiety and depression on cancer patients. Study of K. Zhou et al. (2015) which deliver muscle relaxation and music listening simultaneously for 30 minutes twice a day, once in the early morning (6 a.m-8 a.m.) and once in the evening (9 p.m -11 p.m), after radical mastectomy until discharged from the hospital. The results showed that MCP had a large effect size on anxiety (Cohen’s d= 1.74) and medium effect size on depression (Cohen’s d= 0.72).

Moreover, MCP had a strong effect size on depression in comparison to PMR alone (Liao et al., 2018b). A study by Liao et al. (2018b) illustrated that cancer patients do 20-min PMRT followed by 20-min Chinese medicine five-element music at 10 a.m. or 3 p.m, for 8 weeks could reduce depression level rather than PMR alone ( Cohen’s d= 0.9). Thus, the combination of MI and PMR may boost the effectiveness of single interventions (Liao et al., 2018a)

**Theoretical framework**

Chirico et al. (2020) used the SCT of Lazarus and Folkman (Lazarus, 1984) to guide the study to assess the effectiveness of MI on anxiety and mood state in breast cancer receiving chemotherapy. The results of the study showed a large size effect on anxiety and depression in compassion to the control group. SCT was widely used to test the effectiveness of PMR on issue related stress on cancer patients (Tsitsi et al., 2017). Thus, SCT will be used to underpin this study.

Stress and coping theory focus on the way of people tackle stress, coping is transactions between the individual and the environment. When an individual encounters a situation results in interaction with the environment, the individual will experience an appraisal process to evaluate the stressor which may result in positive or irrelevant effects or harm/loss, threats, challenges. If she/he identify the stressor as harm/loss, threats, challenges, but she/he is not enough available resources, the individual will suffer stress. Then, appropriate coping strategies will be applied to overcome the situation (Lazarus, 1984).

BGC cancer diagnosis and receiving chemotherapy is a potential stressor that may affect psychosocial outcomes such as anxiety, depression, and quality of life. An individual with differences in demographic characteristics (Hulbert-Williams et al., 2012) has various responses to the stressor. Similarly, the environment including the clinical factors affect stress level. MCP will be a coping strategy while family support plays a role as a factor to improve the effectiveness of the intervention.

# Qualitative study

As SCT, appraisal of stress depends on environmental factors including social, culture and different coping methods in the same stressful situation among the different populations (Lazarus, 1984). Besides, music is a cultural factor, the music perception is different. Thus, using music intervention need to be culturally adapted, especially in Vietnam where music intervention have not been widely applied in clinical settings (Fitzsimons, 2016). Thus, a qualitative study was conducted to explore the perception of MI among women with BGC receiving chemotherapy in Vietnam**.**

We interviewed 20 participants. After analysing the data, the preferred music for relaxation was religious music, soft melody music and revolutionary music, while distaste music was fast, strong music and ancient folk songs with very slow melody. The perceptions of music intervention were identified including, perceived beneficial effects, perceived differences in musical preferences of individuals, the feasibility of implementing MI. Family support is the most commonly cited coping measure. Therefore, stress management strategies need to utilize resources from family members, especially husbands. MI has been identified as a beneficial measure to reduce stressful situations. This is also the therapy that is probably acceptable in the vast majority of patients. The musical preferences found in this study could be the foundation for use in subsequent MI trials. Using the patient’s favourite music is more acceptable and probably more effective.

# Intervention development

1. **Theory-guided study development**

The stress and coping theory will be used to develop the intervention protocol.

**Appraisal step:** After finishing baseline assessments, the participant will be arranged to have an individual face-to-face meeting in a private room. The level of anxiety and depression at baseline will be considered. Besides, the intervener will interview the participants a question “How is your emotion recently?”. This will help the intervener know the level of anxiety and depression of the participant. After that, to identify the coping methods, the participants will be asked “what did you do to reduce your stress, recently?”. The intervener will advise participants to keep doing the positive coping methods and avoid the negative coping methods. Based on the result of the qualitative study in phase I, a list of music will be prepared. The questions “what is your favourite music?” “ what type of music that you do not like?” will be asked to participants. The list of patients’ music preferences will be noted to prepare for each participant. The conversation will be recorded and analysed to explain the result of the intervention effectiveness.

**Family support:** Family support is one of the most important coping methods that we identified in the qualitative study. Thus, in our program, the family member will be the person to support and encourage participants to self-practice MI and PMR at home. The family member is the person who lives with the patient and takes care of them every day. They will have an individual face to face meeting with the intervener. They will be informed of the psychological issues of the patients. The intervener will be introduced to the MCP program, its potential benefits. They will assign the task of motivating and monitoring the patient's daily practice.

**Coping strategy:** music listening combine with PMR

**Reappraisal:** Three weekly follow-up callings will be done by a research assistant. The content of the call will be to ask them about their emotion, any issues during practice, any day they cannot practice and the reason. The research assistant will answer questions and encourage them to keep practising. The call will be recorded and analysed.

## Contents and dose of MCP intervention

PMR procedure was described by Bernstein and Borkovec (1973). Following the guideline, 20 minutes practising PMR once/day will be applied. From the literature review, 15-20 minutes of music listening was most effective for cancer patients receiving chemotherapy. Thus, in this study, the length of music will be 20 minutes and PMR will be 20 minutes. Patients will practice once/day (Liao et al., 2018a). Based on the results of the qualitative study, the preferred music may be easier to be accepted, so the patients’music preference will be adopted. The list of music based on the qualitative study including folk songs, religious music, Vietnamese bolero music, and music suggested by the [American Music Therapy Association will be used. The tempo of the music will be 60-80 BPM.](https://www.musictherapy.org/)

**PMR and music preparation**

The PMR that requires the participants to tense and relax 16 groups of muscles covering the face, neck, chest, abdomen and four limbs will be adopted. The content of the PMR script and the voice of the audio-guided will be validated by a panel. The audio guided PMR will be instructed by the intervener’s voice.

The audio taps combine patient’s preferred music with PMR will be created for each individual based on their selected music. To make patients have more music choices for each day. Each patient will have seven different audios with the same initial guide and PMR guide but different music tracks. The audio will be sent to each participant and will be used in the training session for the patient.

1. **MCP Tranining program**

The participants will be trained in the MCP program in a private, quiet room on an individual or group basis. Each group will be not over 5 patients. Participants will be required to wear loose-fitting soft clothes when practising the MCP. After being introduced to the program, the effects of MI and PMR, the patients will be trained on the method of performing PMR and listening to music. The intervener will demonstrate step by step and the participants will return demonstration. The intervener will observe, guide them during the redemonstration. After the training, the intervener will ask participants that having any issues, difficulties during their practice and encourage self-practice daily when they feel the most comfortable at home. To master the skills of the participants at home, colour pictures interpret the steps of the MCP program will distribute to them after the training program.

1. **Content validation of MCP protocol**

A panel of six experts will be invited to validate the MCP protocol. The panel will be two academic professors, two music therapists, two PMR experts. All experts have experience working with cancer patients. First, the researcher will send an email to invite the experts to involve the study. If they agree to participate, the researcher will send the study aim, intervention protocol, script of PMR, audio file of PMR for participant self-practice, assessment form, assessment guideline via email. The audio will be read in Vietnamese, but the expert will focus on evaluating voice tone and speed. The expert will be required to send the feedback after one week. The assessment will rate each content of the protocol by a 4-point Likert scale in which 4= very appropriate, 3= appropriate, 2= Inappropriate, 1= Very inappropriate. The experts will encourage to explain the reason for rating as inappropriate.

# Randomized controlled trial

# Pilot study

Before conducting main study, pilot study will be carried out.

1. **Aim and objectives**

**Aim**

To test the feasibility and acceptability of MCP among BGC patients receiving chemotherapy in Vietnam.

**Objectives**

To evaluate the potential screening, recruitment capability.

To identify potential adverse events associated with MCP.

To preliminary evaluate the effects of MCP on anxiety and depression and QoL.

To estimate the sample size for the future full-scale RCT.

To refine the study protocol for a future full-scale RCT.

1. **Sample size estimation**

Convenience sampling will be used to recruit the participants. The calculated sample size will follow the rules of thumb for finding an appropriate sample size in which the outcome is a continuous measurement. Julious (2005) recommended that for the evaluation feasibility of 2-group studies, a minimum of 12 participants of each group be considered

1. **Methods**

The study procedure, randomization, data collection, data analysis will be similar to the main study (see the main study below).

**Full-Scale study**

1. **Aim and objectives**

**Aim:** To assess the effect of MCP on anxiety, depression, stress and QoL of BGC patients receiving chemotherapy.

**Objectives**

- To assess the effect of MCP on anxiety of BGC patients receiving chemotherapy.
- To assess the effect of MCP on depression of BGC patients receiving chemotherapy.
- To assess the effect of MCP on stress of BGC patients receiving chemotherapy.
- To assess the effect of MCP on QoL and patients’ satisfaction of BGC patients receiving chemotherapy.

**Hypothesis**

- The participants receiving MCP will have a significantly lower level of anxiety compared to the control group.
- The participants receiving MCP will have a significantly lower level of depression compared to the control group.
- The participants receiving MCP will have a significantly lower level of stress compared to the control group
- The participants receiving MCP will have significantly higher QoL scores compared to the control group.

1. **Study design**

A 2-arm, assessor-blinded randomised controlled will be conducted. Randomized Controlled Trial (RCT) is the best design to confirm an objective assessment of the true benefits of medical interventions (Doig & Simpson, 2005). The design will follow the Standard Protocol Items Recommendations for Interventional Trials (SPIRIT) (Chan et al., 2013) .

1. **Participants**

**Inclusion criteria**

- Women with breast or gynaecological cancer are 18 years of age or older, and independent legally to sign the consent form.
- Have a 3-week cycle of chemotherapy regimen and have at least three chemotherapy cycles left.
- Have Karnofsky score (Mor et al., 1984) ≥ 80 ( to be able to self-practice MCP)
- Can communicate, read and write in Vietnamese
- Have a device such as a smartphone, MP3 to keep the audio file (to self-practice MCP at home)
- Consent to join the study.

**Exclusion criteria**

- The patients cannot understand the study procedures
- Have mental health illness, deafness, blindness
- Be participating in other studies related to psychological issues
- Be receiving anxiety or depression treatment.
- Be participating in relaxation therapy such as yoga, meditation, mindfulness, PMR...
- Have a potential treatment (eg. surgery) or personal plans that will prevent them from practising daily during the 6 weeks of the intervention.

1. **Settings**

Participants will be recruited from four public hospitals, Vietnam National Cancer Institute, Hanoi Oncology Hospital in Hanoi, Bach Mai hospital and Nam Dinh general hospital, Vietnam. These hospitals examine and treat patients from the northern and central provinces of Vietnam.

1. **Study sample**

Basing on the study of K. N. Zhou et al. (2015) the effect sizes of anxiety and depression are 1.72 and 0.74, respectively. Thus, the effect size of depression will be used to calculate the sample size. G*power 3.1 software is used with a type 1 error of 5% (α=0.05) and 90% power (β=0.05), two tails, the sample size is 80. The attrition rate in the study of Liao et al. (2018) was 5% after 8 weeks self-practising MCP at home daily. Thus, the final sample size is 84 and each group is 42. The sample size may be adjusted based on the results of the pilot study.

1. **Randomization and allocation concealment**

Blocked randomisation procedures with a 1:1 allocation will be performed by an independent researcher who will not involve any steps of the study. A randomization list will be computer-generated by using the website at https://www.sealedenvelope.com/simple-randomiser/v1/lists. To prevent the investigator from predicting the treatment sequence, a mix of blocks of sizes 4, 6, 8 at random (Efird, 2011) will be used. Besides, blinding both the sequence of blocks and their block size will be applied to be a more effective protection preventing selection bias (Efird, 2011).

Sequentially numbered, opaque, sealed envelopes will be prepared. To avoid tampering the creator of the envelope will sign on the back of the envelope (Clark et al., 2020). In addition, security measures will include inserting carbon paper into the envelopes to prevent trans-illumination (Clark et al., 2020). The person who creates and keeps the envelopes will be blinded and not be involved in recruiting participants to prevent the ordering of participants into groups (Efird, 2011). The envelopes that contain the names of the assigned group will be given to each patient after they sign the consent form. The envelopes will be opened only after the envelope has been irreversibly assigned to the participants (Doig & Simpson, 2005).

1. **Blinding**

It is impossible to blind participants or interveners because of the nature of the study (Huang et al., 2010). However, research assistants who assess outcome data, data entry and

analysis will be blinded to avoid the risk of assessor bias. The assistants will not participate in any other stage of implementing the program. The participants will be asked not to share their treatment allocation with the research assistants.

1. **Outcome measures and instruments**

Primary outcomes will include anxiety and depression, stress while the secondary outcome will encompass QoL.

Participants’ characteristic data will be collected via a self-designed demographic sheet which will include age, marital status, education level, employment, family income, stage of cancer, frequency of chemotherapy, treatment regimen.

Anxiety, depression and stress will be measured using the short form of the Depression Anxiety Stress Scale (DASS-21) (Lovibond & Lovibond, 1995). The instrument was translated and used in the Vietnamese population with good internal consistency (Cronbach’s alpha of depression subscale 0.72; Anxiety subscale 0.77; and Stress subscale 0.70, overall 0.88) (Tran et al., 2013). It includes 21 items rating scale, 7 items for anxiety (DASS21-A), 7 items for depression (DASS21-D) and 7 items for stress (DASS21-S). A 4-point Likert-type scale ranging from 0 to 3 was designed on each item, thus each subscale had a score from 0 to 21. The higher the score is the more severe is the anxiety, depression and stress.

FACT-G will be used to assess QoL. It evaluates in functional scales on Social/family well-being (SWB); Emotional well-being (EWB) and Functional well-being (FWB). Each item is scored in one of five categories ‘Not at all’, ‘A little bit’, ‘some what’, ‘Quite a bit’ ‘Very much’. This tool has been translated and validated in Vietnamese by the FACIT system with the Cronbach’s alpha for the scale being 0.89.

1. **Procedure**

Patients receiving chemotherapy meeting research criteria will be identified by four research assistants who are nurses in the chemotherapy units. The research assistants will contact the participants to obtain written informed consent. If patients agree to participate in the study, they will be randomised to the intervention group or the control group. To assess the recruitment capability, the recruitment rate, the consent rate, and refusal rate, the reason for refusal, ineligibility, number of people signing the consent form will be recorded.

Participants will be assessed at 3-time points by an outcome assessor: after randomization, the baseline (T0) will evaluate the demographic form, DASS-21, FACT-G,.After finishing 3 weeks of self-practice intervention (T1) will assess DASS-21, FACT-G, ,after 3 weeks follow-up (T2) will assess DASS-21, FACT-G. After 6 weeks, the attrition rate will be calculated by recording the participants discontinue or loss of contact, and the reasons for drop out. Three weeks after baseline all the patients will return hospital for the next chemotherapy treatment. In the previous studies, timepoints assessed the effectiveness of MI PMR in cancer patients receiving chemotherapy based on the chemotherapy cycle. Yilmaz and Arslan (2015) evaluated PMR on breast cancer for three weeks (one chemotherapy cycle) and Chen et al. (2020) assess the consistency of MI after three weeks of follow-up. The data of T1 and T2 will be collected during hospitalization by the outcome assessor. The outcome assessor will be trained to collect data. The outcome assessor will be blinded to the study groups to collect data and not involve any steps of the study.

The Intervention Group will receive the MCP training and their family’s member will have a meeting with the intervener. The patients will self-practice once every day. A “Self-practice Record Form (SRF)” will be given to participants to report the adherence, the adverse events, and the reasons for non-adherence. Once every week the research assistant 3 will individually call participants to encourage them to keep practising, remind fill SRF and asked their feelings, emotions. After three weeks of treatment, the participants will be required to fill DASS-21, FACT-G, SRF. After that, a three-week follow up will be carried out to test the persistence effect of MCP. After finishing the follow-up time, the participants will be asked to complete DASS-21, FACT-G, SRF by a research assistant.

The control group will receive standard care including health assessment, regular health advice and nutrition consultation during chemotherapy by doctors and nurses. To minimize the bias by contacting, weekly phone calls also will be carried out to participants. The content of the call will last around two minutes that will ask about their general health, medicine and remind them to return to the hospital next chemotherapy on time.

**Table 3: Time points for data collection**

|  | **Screening** | **Week 0**  **Baseline** | **Week 1-3** | **Week 3** | **Week 6** |
| --- | --- | --- | --- | --- | --- |
| Number of eligible participants | X |  |  |  |  |
| Number of participants sign the consent form | X |  |  |  |  |
| Reason for ineligibility, refusal | X |  |  |  |  |
| DASS-21 |  | X |  | X | X |
| FACT-G |  | X |  | X | X |
| Karnofsky performance status scale | X |  |  |  |  |
| SRF |  |  | X |  |  |
| Process evaluation (interview) |  |  |  | X |  |


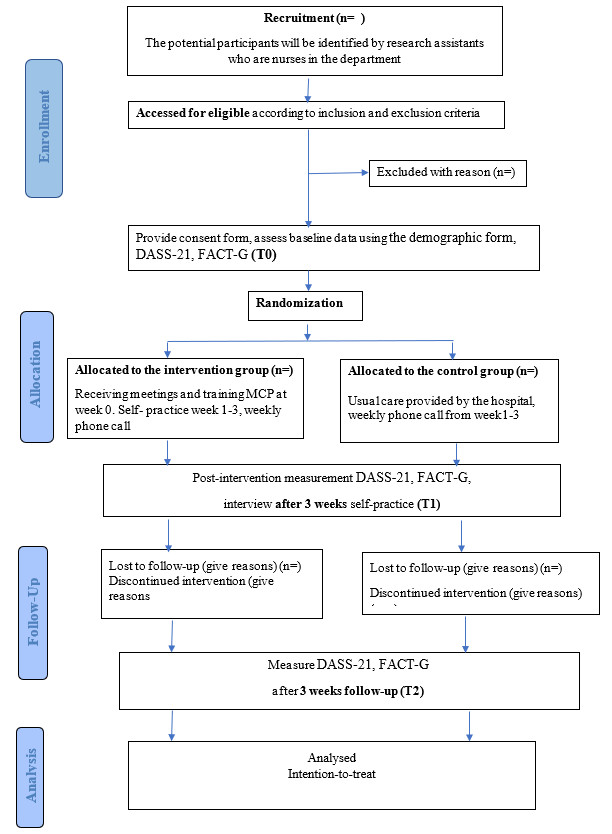


**Fig. 2. The Consolidated Standards of Reporting Trial (CONSORT) flow diagram (Schulz et al., 2010)**

1. **Intervention fidelity**

Contamination will be minimized by asking the participants not to share the intervention information with other patients. The research assistants will be chosen to participate in the study if they have a bachelor’s degree or a higher qualification, a minimum five-year experience in oncology and have research experience. They will be trained in the data collection process, interview skills to ensure consistency. Only one intervener will deliver MCP training to the patients. The intervener was trained and received music therapy and PMR certificates. To ensure adherence to intervention protocol, the written intervention manual, the checklist will be used. To prevent loss of contact, at least two phone numbers of the patient and the patient's family will be saved.

1. **Data analysis**

Statistical analysis SPSS version 25 will be used, and an intention-to-treat analysis will be used in line with the CONSORT statement. The chi-squared test or Fisher’s exact test, wherever appropriate will be used for categorical data (e.g. family income, cancer stage), while the continuous variables (e.g. age) will use the independent T-test to test the homogeneity at baseline between groups. Categorical variables will be reported as frequencies and percentages and continuous variables will be presented as mean± standard deviation. A two-tailed, two-sample T-test (normally distributed) or Mann–Whitney *U* test (non-normally distributed) will be used to compare the mean difference of DASS-21, FACT-G and subscales score of Intervention Group and Control Group between baseline (T0) and T1, T2, respectively. Cohen’s d will be used to measure the intervention effect size on anxiety and depression, stress, QoL of Intervention Group and Control Group. A generalized estimating equation (GEE) model will be used to compare the difference in each outcome across different time points.

**Process evaluation**

Process evaluation study will follow Medical Research Council (MRC) guidance (Moore et al., 2015).

Contextual factors such as family support, caring service will be observed and interview. The feelings, experience of MCP program will be assessed by interview

**Description of intervention and its causal assumption**

**Implementation**

Intervention process (checklist)

Patients’ adherence

(SRF, phone call)

**Mechanisms of impact**

adverse events (SPF)

Participant response to intervention (interview)

Outcomes

**Fig. 3. MRC process evaluation framework (Moore et al., 2015)**

Contextual factors including family support, caring service in the hospital will be observed and documented by using field notes. The family support to practising the program at home, room to practice at home will be interview. Treatment will be documented. The experience of participants after training MCP the intervention will be interviewed and recorded. The intervention process will be recorded by comparing a checklist and an intervention manual. Participant’s adherence, adverse events will be recorded daily by a self-report form (SRF) and phone call follow-up. Participants’ responses will be collected by face-to- face individually interviewing after the intervention participants. Purposeful sampling with maximum variation strategy (Patton, 1990) with various characteristics and levels of adherence will be used. The interview will be recorded and analysed following qualitative content analysis of an interview text described by Graneheim and Lundman (Graneheim & Lundman, 2004).

1. **Ethics**

The study will be subjected to approval from The Joint Chinese University of Hong Kong - New Territories East Cluster Clinical Research Ethics Committee (The Joint CUHK-NTEC CREC) and the Ethics Committee in medical research, Nam Dinh University of Nursing, Vietnam, Vietnamese Ministry of Health.

This study will complies with the Declaration of Helsinki and follow the ethical principles for research that involved human subjects which are: autonomy, non-maleficence, and confidentiality (Beauchamp & Childress, 2001).

**Autonomy**

Written informed consent will be obtained from participants. Participation will voluntary basis and participants could withdraw from the study at any time. In this study, the potential participant will be given an information sheet that includes the purpose, objectives, and study procedures.

**Non-maleficence**

This ethical rule requires the study should not cause any harm to the participants (Beauchamp & Childress, 2001). The component intervention including music listening and PMR was evident as safe non-pharmacological interventions in the previous studies (Bulfone et al., 2009; Holland et al., 1991)

**Confidentiality**

Each participant will be allocated each code and their identity will not be revealed in the findings. Access to code lists for key code is limited to only the main researcher and research assistants. The data files will be kept in a password-protected computer. The paper documents related to the personal information of patients will be kept stored securely in locked cabinets. Consent forms will also be kept securely in locked cabinets, separately from the research data. Contact list, other documents that contain personal information of the participants will be destroyed when no longer required for the research (3 years after finishing data collection).

**Ethical concerns regarding paying participants**

Each participant will be received 100,000 VND (around HK$35) as an honorarium to compensate for the internet fee. Thus, it will not affect any sense of agreement to participate in the study of participants.

**Permission to use the instruments**

All instruments which were used in this study gain approval to use from developers and authors who translated and validated in Vietnamese.

**Registration of the intervention study:** The protocol of the study will be registered on ClinicalTrials.gov.

1. **Study significance**

This is the first study evaluating the effectiveness of the MI and PMR combination on anxiety, depression and QoL in BGC patients receiving chemotherapy. This is also the first study assessing MI and PMR on cancer patients in Vietnam. This study will be culturally adapted by using the patients’ preferred music explored in phase II qualitative study. This is the first theory-based RCT to assess MI and PMR on anxiety, depression and QoL on BCG patients receiving chemotherapy. The theory-driven approach will help to direct the design, variables, outcomes. It will help to understand the mediating process that will contribute to knowing the mechanism of the intervention effects (Sidani & Braden, 1998). The process evaluation will identify the related factors facilitating or impeding the intervention. This will help future studies to adjust to building a more effective design of the intervention. Moreover, the process evaluation will help nurses to identify the most effective condition for their patients in nursing practice. Furthermore, the implementation of MCP to manage psychological disorders will contribute to evidence for nursing practice. It will help to improve the patient's quality of life and increase patient satisfaction with care.

# Study plan

| Contents | Oct-Dec,21 | Jan-Feb22 | Mar- Apr  22 | May- Aug  22 | Sep- Nov  22 | Dec 22- Jun  23 |
| --- | --- | --- | --- | --- | --- | --- |
| Ethical apply (NTEC CREC), Viet Nam |  |  |  |  |  |  |
| Training assistants |  |  |  |  |  |  |
| Pilot study |  |  |  |  |  |  |
| Main RCT data collection |  |  |  |  |  |  |
| Data analysis |  |  |  |  |  |  |
| Complete writing thesis and publications |  |  |  |  |  |  |

**References**

Arakawa, S. (1997, Oct). Relaxation to reduce nausea, vomiting, and anxiety induced by chemotherapy in Japanese patients. *Cancer Nurs, 20*(5), 342-349. <https://doi.org/10.1097/00002820-199710000-00005>

Ballenger, J. C. (2000). Anxiety and depression: optimizing treatments. *Primary Care Companion to the Journal of Clinical Psychiatry, 2*(3), 71.

Beauchamp, T. L., & Childress, J. F. (2001). *Principles of biomedical ethics*. Oxford University Press, USA.

Bernstein, D. A., & Borkovec, T. D. (1973). Progressive relaxation training: A manual for the helping professions.

Bradt, J., Dileo, C., Magill, L., & Teague, A. (2016, Aug 15). Music interventions for improving psychological and physical outcomes in cancer patients. *Cochrane Database of Systematic Reviews*(8), Cd006911. <https://doi.org/10.1002/14651858.CD006911.pub3>

Bro, M. L., Johansen, C., Vuust, P., Enggaard, L., Himmelstrup, B., Mourits-Andersen, T., Brown, P., d’Amore, F., Andersen, E. A. W., Abildgaard, N., & Gram, J. (2019). Effects of live music during chemotherapy in lymphoma patients: a randomized, controlled, multi-center trial [Article]. *Supportive Care in Cancer, 27*(10), 3887-3896. <https://doi.org/10.1007/s00520-019-04666-8>

Bulfone, T., Quattrin, R., Zanotti, R., Regattin, L., & Brusaferro, S. (2009, Jul-Aug). Effectiveness of music therapy for anxiety reduction in women with breast cancer in chemotherapy treatment. *Holistic Nursing Practice, 23*(4), 238-242. <https://doi.org/10.1097/HNP.0b013e3181aeceee>

Burns, D. S., Azzouz, F., Sledge, R., Rutledge, C., Hincher, K., Monahan, P. O., & Cripe, L. D. (2008). Music imagery for adults with acute leukemia in protective environments: a feasibility study. *Supportive Care in Cancer, 16*(5), 507‐513. <https://doi.org/10.1007/s00520-007-0330-z>

Burrai, F., Micheluzzi, V., & Bugani, V. (2014). Effects of live sax music on various physiological parameters, pain level, and mood level in cancer patients: a randomized controlled trial. *Holistic Nursing Practice, 28*(5), 301-311.

Chan, A.-W., Tetzlaff, J. M., Altman, D. G., Laupacis, A., Gøtzsche, P. C., Krleža-Jerić, K., Hróbjartsson, A., Mann, H., Dickersin, K., Berlin, J. A., Doré, C. J., Parulekar, W. R., Summerskill, W. S. M., Groves, T., Schulz, K. F., Sox, H. C., Rockhold, F. W., Rennie, D., & Moher, D. (2013). SPIRIT 2013 statement: defining standard protocol items for clinical trials. *Annals of Internal Medicine, 158*(3), 200-207. <https://doi.org/10.7326/0003-4819-158-3-201302050-00583>

Chen, S. C., Yeh, M. L., Chang, H. J., & Lin, M. F. (2020, Jan). Music, heart rate variability, and symptom clusters: a comparative study. *Support Care Cancer, 28*(1), 351-360. <https://doi.org/10.1007/s00520-019-04817-x>

Chirico, A., Maiorano, P., Indovina, P., Milanese, C., Giordano, G. G., Alivernini, F., Iodice, G., Gallo, L., De Pietro, G., Lucidi, F., Botti, G., De Laurentiis, M., & Giordano, A. (2020, 01 Jun). Virtual reality and music therapy as distraction interventions to alleviate anxiety and improve mood states in breast cancer patients during chemotherapy. *Journal of Cellular Physiology, 235*(6), 5353-5362.

Clark, L., Dean, A., Mitchell, A., & Torgerson, D. J. (2020). Envelope use and reporting in randomised controlled trials: A guide for researchers. *Research Methods in Medicine & Health Sciences*, 2632084320957204.

Craig, P., Dieppe, P., Macintyre, S., Michie, S., Nazareth, I., & Petticrew, M. (2013, 2013/05/01/). Developing and evaluating complex interventions: The new Medical Research Council guidance. *International Journal of Nursing Studies, 50*(5), 587-592. <https://doi.org/https://doi.org/10.1016/j.ijnurstu.2012.09.010>

Dikmen, H. A., & Terzioglu, F. (2019, Feb). Effects of Reflexology and Progressive Muscle Relaxation on Pain, Fatigue, and Quality of Life during Chemotherapy in Gynecologic Cancer Patients. *Pain Manag Nurs, 20*(1), 47-53. <https://doi.org/10.1016/j.pmn.2018.03.001>

Doig, G. S., & Simpson, F. (2005, 2005/06/01/). Randomization and allocation concealment: a practical guide for researchers. *Journal of Critical Care, 20*(2), 187-191. <https://doi.org/10.1016/j.jcrc.2005.04.005>

Efird, J. (2011). Blocked randomization with randomly selected block sizes. *International journal of environmental research and public health, 8*(1), 15-20.

Ell, K., Sanchez, K., Vourlekis, B., Lee, P.-J., Dwight-Johnson, M., Lagomasino, I., Muderspach, L., & Russell, C. (2005). Depression, correlates of depression, and receipt of depression care among low-income women with breast or gynecologic cancer. *Journal of clinical oncology : official journal of the American Society of Clinical Oncology, 23*(13), 3052-3060. <https://doi.org/10.1200/JCO.2005.08.041>

Ferlay J, Ervik M, Lam F, Colombet M, Mery L, Piñeros M, Znaor A, Soerjomataram I, & Bray F. (2020). *Global Cancer Observatory: Cancer Today*. Lyon, France: International Agency for Research on Cancer. Retrieved 17 May from <https://gco.iarc.fr/today>

Ferrer, A. J. (2007). The effect of live music on decreasing anxiety in patients undergoing chemotherapy treatment. *J. Music Ther, 44*(3), 242‐255. <https://doi.org/10.1093/jmt/44.3.242>

Fitzsimons, B. (2016, 2016/11/01). Approaching music therapy in a different country: A literature review on cultural considerations when practising in a developing country. *British Journal of Music Therapy, 30*(2), 83-88. <https://doi.org/10.1177/1359457516667930>

Garmany, G. (1952). *Muscle relaxation as an aid to psychotherapy*. Actinic Press.

Gok Metin, Z., Karadas, C., Izgu, N., Ozdemir, L., & Demirci, U. (2019, Oct). Effects of progressive muscle relaxation and mindfulness meditation on fatigue, coping styles, and quality of life in early breast cancer patients: An assessor blinded, three-arm, randomized controlled trial. *Eur J Oncol Nurs, 42*, 116-125. <https://doi.org/10.1016/j.ejon.2019.09.003>

Graneheim, U. H., & Lundman, B. (2004, 2004/02/01/). Qualitative content analysis in nursing research: concepts, procedures and measures to achieve trustworthiness. *Nurse Education Today, 24*(2), 105-112. <https://doi.org/https://doi.org/10.1016/j.nedt.2003.10.001>

Herizchi, S., Asvadi, I., Piri, I., Golchin, M., Shabanlui, R., & Sanaat, Z. (2012). Efficacy of Progressive Muscle Relaxation Training on Anxiety, Depression and Quality of Life in Cancer Patients Undergoing Chemotherapy at Tabriz Hematology and Oncology Research Center, Iran in 2010. *Middle east journal of cancer, 3*(1), 9-13. <http://search.ebscohost.com/login.aspx?direct=true&db=ccm&AN=104498973&site=ehost-live&scope=site>

Hoang, H. T. X., Molassiotis, A., Chan, C. W., Nguyen, T. H., & Liep Nguyen, V. (2020, 2020/03/01). New-onset insomnia among cancer patients undergoing chemotherapy: prevalence, risk factors, and its correlation with other symptoms. *Sleep and Breathing, 24*(1), 241-251. <https://doi.org/10.1007/s11325-019-01839-x>

Holland, J. C., Morrow, G. R., Schmale, A., Derogatis, L., Stefanek, M., Berenson, S., Carpenter, P. J., Breitbart, W., & Feldstein, M. (1991, 1991/06/01). A randomized clinical trial of alprazolam versus progressive muscle relaxation in cancer patients with anxiety and depressive symptoms. *Journal of Clinical Oncology, 9*(6), 1004-1011. <https://doi.org/10.1200/JCO.1991.9.6.1004>

Huang, S., Good, M., & Zauszniewski, J. A. (2010). The effectiveness of music in relieving pain in cancer patients: A randomized controlled trial. *International journal of nursing studies, 47*(11), 1354-1362. <https://doi.org/10.1016/j.ijnurstu.2010.03.008>

Hulbert-Williams, N., Neal, R., Morrison, V., Hood, K., & Wilkinson, C. (2012, Aug). Anxiety, depression and quality of life after cancer diagnosis: what psychosocial variables best predict how patients adjust? *Psycho-Oncology, 21*(8), 857-867. <https://doi.org/10.1002/pon.1980>

Jasemi, M., Aazami, S., & Zabihi Roghaieh, E. (2016). The Effects of Music Therapy on Anxiety and Depression of Cancer Patients. *Indian journal of palliative care, 22*(4), 455‐458.

Julious, S. A. (2005, 2005/10/01). Sample size of 12 per group rule of thumb for a pilot study [<https://doi.org/10.1002/pst.185>]. *Pharmaceutical Statistics, 4*(4), 287-291. <https://doi.org/https://doi.org/10.1002/pst.185>

Lazarus, R. S. (1984). *Stress, appraisal, and coping*. Springer Pub. Co.

Lerman, C., Rimer, B., Blumberg, B., Cristinzio, S., Engstrom, P. F., MacElwee, N., O'Connor, K., & Seay, J. (1990, Oct). Effects of coping style and relaxation on cancer chemotherapy side effects and emotional responses. *Cancer Nurs, 13*(5), 308-315.

Liao, J., Wu, Y., Zhao, Y., Zhao, Y. C., Zhang, X., Zhao, N., Lee, C. G., & Yang, Y. F. (2018a, May). Progressive Muscle Relaxation Combined with Chinese Medicine Five-Element Music on Depression for Cancer Patients: A Randomized Controlled Trial. *Chin J Integr Med, 24*(5), 343-347. <https://doi.org/10.1007/s11655-017-2956-0>

Lima, T. U., Moura, E. C. R., Oliveira, C. M. B. d., Leal, R. J. D. C., Nogueira Neto, J., Pereira, E. C., Nascimento, R. V. B., Oliveira, E. J. S. G. d., & Leal, P. d. C. (2020). Impact of a Music Intervention on Quality of Life in Breast Cancer Patients Undergoing Chemotherapy: A Randomized Clinical Trial. *Integrative Cancer Therapies, 19*, 1534735420938430.

Lin, M.-F., Hsieh, Y.-J., Hsu, Y.-Y., Fetzer, S., & Hsu, M.-C. (2011). A randomised controlled trial of the effect of music therapy and verbal relaxation on chemotherapy-induced anxiety. *J Clin Nurs. Vol.20(7-8), 2011, pp. 988-999.*

Lovibond, S., & Lovibond, P. (1995). Manual for the depression anxiety stress scales. 2nd edn. Sydney: Psychology Foundation, 1995. *Google Scholar*, 4-42.

Miaskowski, C., Barsevick, A., Berger, A., Casagrande, R., Grady, P. A., Jacobsen, P., Kutner, J., Patrick, D., Zimmerman, L., & Xiao, C. (2017). Advancing symptom science through symptom cluster research: Expert panel proceedings and recommendations. *JNCI: Journal of the National Cancer Institute, 109*(4).

Mondanaro, J. F., Sara, G. A., Thachil, R., Pranjic, M., Rossetti, A., EunHye Sim, G., Canga, B., Harrison, I. B., & Loewy, J. V. (2020). The Effects of Clinical Music Therapy on Resiliency in Adults Undergoing Infusion: A Randomized, Controlled Trial. *Journal of Pain and Symptom Management*. <https://doi.org/http://dx.doi.org/10.1016/j.jpainsymman.2020.10.032>

Moore, G. F., Audrey, S., Barker, M., Bond, L., Bonell, C., Hardeman, W., Moore, L., O’Cathain, A., Tinati, T., Wight, D., & Baird, J. (2015). Process evaluation of complex interventions: Medical Research Council guidance. *BMJ : British Medical Journal, 350*, h1258. <https://doi.org/10.1136/bmj.h1258>

Mor, V., Laliberte, L., Morris, J. N., & Wiemann, M. (1984, 1984/05/01). The Karnofsky performance status scale: An examination of its reliability and validity in a research setting [<https://doi.org/10.1002/1097-0142(19840501)53:9><2002::AID-CNCR2820530933>3.0.CO;2-W]. *Cancer, 53*(9), 2002-2007. <https://doi.org/https://doi.org/10.1002/1097-0142(19840501)53:9><2002::AID-CNCR2820530933>3.0.CO;2-W

Nguyen, A. D., Hoang, M. V., & Nguyen, C. C. (2018, 2018/04/03). Medical costs for the treatment of cervical cancer at central hospitals in Vietnam. *Health Care for Women International, 39*(4), 442-449. <https://doi.org/10.1080/07399332.2017.1402912>

Patton, M. Q. (1990). *Qualitative evaluation and research methods*. SAGE Publications, inc.

Ramezani, T. (2001). Degree of depression and the need for counseling among women with breast cancer in Kerman chemotherapeutic centers.

Schulz, K. F., Altman, D. G., Moher, D., & the, C. G. (2010, 2010/03/24). CONSORT 2010 Statement: updated guidelines for reporting parallel group randomised trials. *BMC Medicine, 8*(1), 18. <https://doi.org/10.1186/1741-7015-8-18>

Schwarz, R., Krauss, O., Höckel, M., Meyer, A., Zenger, M., & Hinz, A. (2008). The Course of Anxiety and Depression in Patients with Breast Cancer and Gynaecological Cancer. *Breast Care, 3*(6), 417-422. <https://doi.org/10.1159/000177654>

Shakeri, J., Golshani, S., Jalilian, E., Farnia, V., Nooripour, R., Alikhani, M., & Yaghoobi, K. (2009). Frequency of depression in patients with breast cancer referring to chemotherapy centers of Kermanshah educational centers (2007-2008). *Asian Pacific Journal of Cancer Prevention, 27*, 324-328.

Shakeri, J., Golshani, S., Jalilian, E., Farnia, V., Nooripour, R., Alikhani, M., & Yaghoobi, K. (2016). Studying the amount of depression and its role in predicting the quality of life of women with breast cancer. *Asian Pacific Journal of Cancer Prevention, 17*(2), 643-646.

Sidani, S., & Braden, C. J. (1998). *Evaluating nursing interventions: A theory-driven approach*. Sage.

Song, Q. H., Xu, R. M., Zhang, Q. H., Ma, M., & Zhao, X. P. (2013). Relaxation training during chemotherapy for breast cancer improves mental health and lessens adverse events. *Int J Clin Exp Med, 6*(10), 979-984.

Tascilar, M., de Jong, F. A., Verweij, J., & Mathijssen, R. H. (2006). Complementary and alternative medicine during cancer treatment: beyond innocence. *The oncologist, 11*(7), 732-741.

Thoma, M. V., La Marca, R., Brönnimann, R., Finkel, L., Ehlert, U., & Nater, U. M. (2013). The effect of music on the human stress response. *PloS One, 8*(8), e70156-e70156. <https://doi.org/10.1371/journal.pone.0070156>

Tran, T. D., Tran, T., & Fisher, J. (2013, 2013/01/12). Validation of the depression anxiety stress scales (DASS) 21 as a screening instrument for depression and anxiety in a rural community-based cohort of northern Vietnamese women. *BMC Psychiatry, 13*(1), 24. <https://doi.org/10.1186/1471-244X-13-24>

Truong, D. V., Bui, Q. T. T., Nguyen, D. T., & Moore, J. (2019). Anxiety among inpatients with cancer: findings from a hospital-based cross-sectional study in Vietnam. *Cancer Control, 26*(1), 1073274819864641.

Tsitsi, T., Charalambous, A., Papastavrou, E., & Raftopoulos, V. (2017, 2017/02/01/). Effectiveness of a relaxation intervention (progressive muscle relaxation and guided imagery techniques) to reduce anxiety and improve mood of parents of hospitalized children with malignancies: A randomized controlled trial in Republic of Cyprus and Greece. *European Journal of Oncology Nursing, 26*, 9-18. <https://doi.org/https://doi.org/10.1016/j.ejon.2016.10.007>

Tuinmann, G., Preissler, P., Bohmer, H., Suling, A., & Bokemeyer, C. (2017). *The effects of music therapy in patients with high-dose chemotherapy and stem cell support: A randomized pilot study*. Psychooncology. Vol.26(3), 2017, pp. 377-384.

Van Minh, H., My, N. T. T., & Jit, M. (2017, May 15). Cervical cancer treatment costs and cost-effectiveness analysis of human papillomavirus vaccination in Vietnam: a PRIME modeling study. *BMC Health Serv Res, 17*(1), 353. <https://doi.org/10.1186/s12913-017-2297-x>

Yilmaz, S. G., & Arslan, S. (2015). Effects of progressive relaxation exercises on anxiety and comfort of Turkish breast cancer patients receiving chemotherapy. *Asian Pac J Cancer Prev, 16*(1), 217-220. <https://doi.org/10.7314/apjcp.2015.16.1.217>

Zhou, K., Li, X., Li, J., Liu, M., Dang, S., Wang, D., & Xin, X. (2015). A clinical randomized controlled trial of music therapy and progressive muscle relaxation training in female breast cancer patients after radical mastectomy: results on depression, anxiety and length of hospital stay. *Eur J Oncol Nurs, 19*(1), 54-59. <https://doi.org/10.1016/j.ejon.2014.07.010>

Zhou, K. N., Li, X. M., Li, J., Liu, M., Dang, S. N., Wang, D. L., & Xin, X. (2015, Feb). A clinical randomized controlled trial of music therapy and progressive muscle relaxation training in female breast cancer patients after radical mastectomy: Results on depression, anxiety and length of hospital stay. *European journal of oncology nursing, 19*(1), 54-59. <https://doi.org/10.1016/j.ejon.2014.07.010>
