# Supplementary material for: Effects of music intervention combined with progressive muscle relaxation on anxiety, depression, stress and quality of life among women with cancer receiving chemotherapy: A pilot randomized controlled trial
Source: PLoS One. 2023 Nov 3;18(11):e0293060. doi: 10.1371/journal.pone.0293060 (PMC10624313; doi:10.1371/journal.pone.0293060)
Supplement: Attachment — Submitted filename: Manuscript (1).docx [file pone.0293060.s004.docx]

**Title:** Effects of Music Intervention combined with Progressive Muscle Relaxation on Anxiety, Depression, Stress and Quality of Life among Women with cancer Receiving Chemotherapy: A Pilot Randomized Controlled Trial

**Short Title:** Music Intervention combined with Progressive Muscle Relaxation on Psychological Issues among Women with Cancer

**Authors:**

Khanh T NGUYEN^1,2^, Huong TX HOANG^3^, Quang V BUI^4^, Dorothy NS CHAN^1^, Carmen WH CHAN^1^*

^1^ The Nethersole School of Nursing, The Chinese University of Hong Kong, Hong Kong SAR, China

^2^ Nam Dinh University of Nursing, Nam Dinh, Vietnam

^3^ Phenikaa University, Hanoi, Vietnam

^4^ Hanoi Oncology Hospital, Hanoi, Vietnam

**Contact detail**

**Contact information**

*Corresponding author: Dr Carmen WH Chan, RN, B.N., MN, PhD

**Address:** Rm732, 7/F, Esther Lee Building, The Chinese University of Hong Kong, Shatin, NT, Hong Kong.

**Email:** [whchan@cuhk.edu.hk](mailto:whchan@cuhk.edu.hk)

**ORCID:** 0000-0003-0696-2369

**Telephone:** (852)39436218

**Email for authors**

Khanh T NGUYEN: [khanhnguyen@link.cuhk.edu.hk](mailto:khanhnguyen@link.cuhk.edu.hk); ORCID: 0000-0002-0332-9309

Huong TX HOANG: [huong.hoangthixuan@phenikaa-uni.edu.vn](mailto:huong.hoangthixuan@phenikaa-uni.edu.vn), ORCID: 0000-0002-5248-0398

Quang V BUI: quangbv@bvubhn.vn

Dorothy NS CHAN: [dorothycns@cuhk.edu.hk](mailto:dorothycns@cuhk.edu.hk), ORCID: 0000-0001-6848-9947

**Abstract**

**Aim:** to investigate the feasibility, acceptability and preliminary effects of music intervention combined with progressive muscle relaxation on anxiety, depression, stress, and quality of life among breast and gynaecological cancer patients receiving chemotherapy

**Methods:** A single-blinded randomized controlled trial was conducted among 24 patients. The intervention group (n=12) received a face-to-face training program about music listening and progressive muscle relaxation skills. They then performed the self-practice daily at home for three weeks. The control group (n=12) received standard care, including health assessment, regular health advice and nutrition consultation. A set of structured questionnaires was administered to collect the data at baseline (T0), post-intervention (3^rd^ week, T1) and follow-up (6^th^ week, T2).

**Results:** A total of 24 patients were eligible to join, and 20 completed the study. Anxiety level significantly decreased at T1 (p=0.02) with a medium effect size (g= 0.62). Although the results failed to show statistically significant effects on depression, stress, and quality of life at both T1, and T2 (p>0.05), the findings illustrated a trend-forward for a larger reduction in depression and stress and improvement in quality of life in the intervention group than in the control group.

**Conclusions:** Implementing music intervention combined with progressive muscle relaxation is feasible and improves anxiety levels. A larger scale randomized controlled trial is needed to reveal the effect of intervention on depression, stress, and quality of life.

**Keywords**

Music intervention, progressive muscle relaxation, psychology, cancer, chemotherapy

**Trial Registration**

This trial was registered on ClinicalTrials.gov with ID: NCT05262621.

1. **Introduction**

Breast and gynaecological cancers are the most common cancers among women in the world [1]. In 2020, 2.3 million new breast cancer cases were estimated, which account for 24.5% of female cancer patients globally. Nearly 1.4 million new gynaecological cancer cases in the same year, including cervix uteri, corpus uteri, ovary, vulva and vagina was reported [2]. Breast and gynaecological cancers caused 1.35 million deaths worldwide in 2020 [2].

Various treatments were introduced, such as surgery, radiotherapy, hormone therapy, and targeted therapy, yet chemotherapy has played a crucial role in breast and gynaecological cancer treatment. Most cancer chemotherapy methods revolve around the idea that cytotoxic drugs can be used to stop proliferating cancer cells [3]. Chemotherapy treatment of cancer has four main goals: curative chemotherapy, adjuvant chemotherapy, neoadjuvant chemotherapy, and palliative chemotherapy [4]. However, cancer patients receiving chemotherapeutic treatments routinely suffer from distressing physical symptoms such as gastrointestinal problems, fatigue, neurological effects [5] and psychological symptoms, including anxiety and depression, which decrease their quality of life (QoL) [6]. It was reported that 26.9% and 41.55 % of breast cancer patients taking chemotherapy experienced anxiety and depression, respectively [7]. A significant prevalence of cases of anxiety (38%) and depression (33%) was found in ovarian cancer patients at the end of chemotherapy [8]. Thus, psychological support for breast and gynaecological cancer patients is essential in nursing care.

To manage these psychological issues, pharmacological and non-pharmacological treatments have been applied. Although pharmacological intervention showed an effect in controlling psychological issues, it can cause various side effects such as nausea, dizziness, diarrhoea, and headaches [9]. Moreover, the drug-to-drug interactions and the cost of treatment should be considered [9]. Non-pharmacological interventions have been introduced to minimize the adverse psychological effects during cancer treatment. Many non-pharmacological interventions were suggested to reduce chemotherapy-related stress, such as cognitive behavioural therapy, problem-solving, yoga [10], distraction, music intervention (MI), virtual reality [11], and progressive muscle relaxation (PMR) [12-14]. Among these interventions, MI and PMR are more feasible to implement than others because they are inexpensive, safe and acceptable [14-17].

A systematic review indicated that MI was an effective method to relieve anxiety and improve cancer patients' QoL during chemotherapy [18, 19]. Especially, anxiety and depression reduction were observed among breast cancer patients taking chemotherapy after receiving MI [16, 18, 20]. PMR is also commonly used for stress management, significantly reducing psychological stress, including anxiety, in breast cancer patients receiving chemotherapy [13, 14].

Many studies have evaluated the effect of MI or PMR as a single intervention, but limited studies evaluated their combined effects on patients’ psychological wellbeing. A combination of MI and PMR intervention was assessed in breast cancer patients after radical mastectomy by Zhou, Li [21] which reported a significant effect of the combined intervention on anxiety and depression. Music combined with PMR has a more significant impact on anxiety, relaxation, and stress than music and PMR alone [22-24]. However, to our knowledge, no study assessed the effect of MI combined with PMR on anxiety, depression and QoL in breast and gynaecological cancer patients receiving chemotherapy. Thus, the objectives of this study are:

- To test the feasibility and acceptability of MI combined with PMR in breast and gynaecological cancer patients receiving chemotherapy.

- To evaluate the preliminary effects of this combined intervention on anxiety, depression, stress, and QoL among breast and gynaecological cancer patients receiving chemotherapy.

2. **Methods**

The study protocol and CONSORT checklist are available as supporting information (see S1 CONSORT Checklist and S2 Study protocol).

2.1. **Design**

A 2-arm, assessor-blinded pilot randomized controlled trial was conducted at a chemotherapy Unit in Hanoi Oncology hospital, Hanoi, Vietnam, from March to May 2022.

2.2. **Participants**

The inclusion criteria were: (1) women with breast or gynaecological cancer aged 18 years or older; (2) receiving chemotherapy once in every three weeks and have at least three chemotherapy cycles left; (3) Have Karnofsky score ≥ 80 [25]; (4) able to communicate, read and write in Vietnamese; (5) have a device such as a smartphone, MP3 to keep the audio file; (6) consent to join the study. We excluded: (1) the patients who cannot understand the study procedures; (2) with mental health illnesses, deafness, or blindness; (3) receiving other treatments or therapy to manage anxiety or depression, (4) with a potential treatment (e.g. surgery) or personal plans that prevent them from practising daily during the six weeks of the intervention.

2.3. **Study sample**

Convenience sampling was used to recruit the participants. The calculated sample size followed the rules of thumb for finding an appropriate sample size in which the outcome is a continuous measurement. Julious [26] recommended that for the evaluation of a feasibility study with 2-arms, a minimum of 12 participants in each arm should be considered.

2.4. **Randomization and allocation concealment**

Blocked randomization procedures with a 1:1 allocation were performed by an independent researcher who did not involve any steps of the intervention program. Sequentially numbered, opaque, sealed envelopes were used. To avoid tampering, the creator of the envelopes signed on the back of the envelopes [27]. The person who prepared and kept the envelopes was blinded and not involved in recruiting participants to prevent ordering participants into groups [28]. Patients receiving chemotherapy and meeting the inclusion criteria were identified by the research assistant. The research assistant contacted the participants. Patients who agreed to participate in the study signed the consent form. They were then asked to complete the baseline measurements before randomization.

**2.5. The combined intervention PMR and MI procedure**

The participants in the intervention group were required to wear loose-fitting, soft clothes, and entered a private, quiet, dimmed light room to receive a face-to-face training session of PMR combined with MI. The training session was performed by the principal investigator (PI), who is certified in PMR and MI. After being introduced to the program, the effects of MI and PMR on psychological issues, the patients were trained on PMR skills and listening to music. The PI demonstrated step-by-step instructions on PMR, and then the participants performed the return demonstration. The participants were instructed on tensing and relaxing 16 muscle groups in sequence, following face and neck, arms and hands, chest and abdomen, legs and feet. Each muscle group tightened for five to seven seconds and then suddenly relaxed for 20-30 seconds. Each muscle group was performed twice. After training PMR skills, the intervener asked participants to select their preferred music from a list including folk songs, religious music, revolutionary music and Vietnamese bolero music. These music were selected based on the findings of a qualitative study conducted by K. T.Nguyen et al. for the types of music preferred by breast and gynaecological cancer patients. [29]. The researcher pre-prepared the list of music tracks with relaxing music with slow, consistent rhythmic, soft melody [18] and a tempo of 60 to 80 beats/min [16, 20, 30]. Instrumental music was used because it was more effective in reducing anxiety than vocal music [31]. After selecting the preferred music, the PI turned on the MP3 file consisting of 20 minutes of PMR instruction and, right after that, 20 minutes the patient's selected music. The participants were asked to lie down, close their eyes, listen to the recorded instruction in the MP3 file and self-practice under the supervision of PI. After the training, the PI asked participants about any issues or difficulties during their practice and encouraged them to self-practice once a day when they feel the most comfortable at home. The patients’ selected MP3 files were sent to their phone via the Zalo application. To remind the skills for the participants at home, a structured guideline and coloured pictures presenting the steps of the PMR were distributed to them. A "Self-practice Record Form (SRF)" was given to participants to report their adherence, any adverse events, and the reasons for non-adherence. The PI arranged a meeting with a family member to explain the benefits of the intervention and assigned them to remind and encourage the participants to practice every day at home. Once every week, a research assistant called participants to encourage them to keep practising, remind filling SRF daily and asked their feelings about the practice.

The control group received standard care, including health assessment, regular health advice and nutrition consultation during chemotherapy by doctors and nurses. To minimize the bias by contacting, weekly phone calls were also carried out to participants to ask about their general health and remind them to return to the hospital in subsequent chemotherapy on time.

**2.6. Outcome measures and instruments**

The recruitment rate, eligibility, consent rate, refusal rate, reasons for refusal, and attrition rate were recorded to assess the feasibility. Individual face-to-face semi-structured interviews were conducted with ten participants in the intervention group to review the acceptability of the intervention. The interview lasted 20 to 30 minutes which covered four questions:

1. Could you describe your experience after three weeks of practising the intervention?
2. How did you feel after practising?
3. Could you give me some comments or suggestions about the program?
4. Would you want to keep practising the intervention in the future?

Participants' demographic characteristic was collected using a demographic sheet which included age, marital status, education level, employment, family income, stage of cancer, frequency of chemotherapy, and treatment regimen.

Anxiety, depression and stress were measured using the short form of the Depression Anxiety Stress Scale (DASS-21) [32]. The instrument was translated and used in the Vietnamese population with good internal consistency (Cronbach's alpha: depression subscale-0.72; Anxiety subscale-0.77; and Stress subscale-0.70, overall scale-0.88) [33]. The scale includes 21 items, with seven items for Anxiety (DASS21-A), seven items for depression (DASS21-D) and seven items for stress (DASS21-S). Each item was measured on a 4-point Likert-type scale ranging from 0= “Do not apply to me at all” to 3= “Applied to me very much or most of the time”. Scores on the DASS-21 needed to be multiplied by 2 to calculate the final score. The score of each subscale ranged from 0 to 42. The higher the score is, the more severe is the anxiety, depression and stress level.

The Functional Assessment of Cancer Therapy – General (FACT-G) was a 27-item self-report measure used to assess QoL in cancer patients. This tool has been translated into Vietnamese. It evaluates functional scales on physical, social, emotional, and functional well-being. Each item is scored in five categories from 0 to 4, meaning 'Not at all, 'A little bit', 'some what', 'Quite a bit' 'Very much'. A total FACT-G score was computed by adding subscale scores. The score ranged from 0 to 108; the higher the score, the better the QOL.

**2.7. Data collection**

The baseline data was collected using the demographic sheet, DASS, and FACT-G by an outcome assessor. The outcome assessor was blinded to the study groups to collect data and did not involve any study steps. DASS, and FACT-G were re-evaluated after finishing three weeks of self-practice intervention (T1) and three weeks after T1 (T2). The interview to review the acceptability of the intervention was conducted at T1 (see Table 1).

**Table 1. Time points for data collection.**

|  | **Screening** | **Week 0**  **Baseline** | **Week 1-3** | **Week 3** | **Week 6** |
| --- | --- | --- | --- | --- | --- |
| Number of eligible participants | X |  |  |  |  |
| Number of participants sign the consent form | X |  |  |  |  |
| Reason for ineligibility, refusal | X |  |  |  |  |
| Karnofsky performance status scale | X |  |  |  |  |
| DASS-21 |  | X |  | X | X |
| FACT-G |  | X |  | X | X |
| SRF |  |  | X |  |  |

**2.8. Data analysis**

Statistical analyses were performed using SPSS version 25. The chi-squared test or Fisher's exact test, wherever appropriate, was used for categorical data (e.g., family income, cancer stage), while the continuous variables (e.g., age) were used Mann-Whitney U to test the homogeneity at baseline between groups. Categorical variables were reported as frequencies and percentages, and continuous variables were presented as mean± standard deviation. Mann–Whitney U test was used to compare the mean difference of DASS and FACT-G between groups at baseline (T0) and T1, T2, respectively. Hedges'g was used to measure the intervention effect size on outcomes for more appropriateness in a small sample size study [34]. The level of statistical significance was set at p <0.05. For the qualitative data, the interview transcriptions were analyzed following a qualitative content analysis approach.

2.9. **Ethical approval**

The pilot study was conducted in accordance with the Declaration of Helsinki**.** The study was approved by The Joint Chinese University of Hong Kong - New Territories East Cluster Clinical Research Ethics Committee (No. 2021.725), the Ethics Committee in medical research, Nam Dinh University of Nursing, Vietnam, Vietnamese Ministry of Health (No. 2792). Written consent was obtained from all individual participants included in the study.

3. **Result**
   3.1. **Feasibility of the study**

The whole data collection process was done from 8^th^ March to 2^nd^ May 2022, in which the total time for recruitment was 14 days. During the recruitment period, 126 patients were assessed for eligibility, of which 94 (74.6%) patients were excluded because of ineligibility. After excluding eight patients with reasons: positive with Covid-19 (one patient), refused (three patients), had Karnofsky score lower than 80 (two patients), and could not arrange time for the intervention (two patients). The remaining 24 consented participants were randomized into the intervention group (n=12) or the control group (n=12).

One participant dropped out (4.2%) at T1 because she changed to another hospital. Three participants dropped out at T2 (12.5%), including two participants in the intervention group due to changing the chemotherapy schedule and feeling exhausted and one in the control group because of the changing chemotherapy schedule (see details in Fig 1).

**Fig 1.** **The Consolidated Standards of Reporting Trial (CONSORT) flow diagram.**

3.2. **Preliminary effects of MI combined with PMR on outcome measures**
     3.2.1 **Participants' demographic and clinical characteristics**

The mean age of the participants was 52.29 years. Nearly half of the participants (45.8%) obtained a bachelor's or higher degree. The majority of participants did not have a religious belief (83.3%). More than half of the participants were current workers (58.3%), living in a city area (66.7%), and married (62.5%). The proportion of people with an average income/ person/ month lower than 210 USD accounted for 54.2%. There was no difference in demographic characteristics between the two groups (see Table 2).

**Table 2. Baseline demographic information of the participants by group assignment.**

| **Variables** | **Total (N=24)** | **Group assignment** | | **p** |
| --- | --- | --- | --- | --- |
|  |  | **MCP group**  **(n=12)** | **Control group (n=12)** |  |
| **Age** (years) Mean (SD)  [Range] | 52.29 (13.44)  [31-75] | 49 (11.75)  [31-74] | 55.58(14.69)  [33-75] | 0.27^a^ |
| **Education** | | | | |
| Secondary school and lower | 9 (37.5%) | 4 (33.3%) | 5 (41.7%) | 0.57^b^ |
| High, vocational school | 4 (16.7%) | 1 (8.3 %) | 3 (25 %) |  |
| Bachelor or higher | 11 (45.8%) | 7 (58.3%) | 4 (33.3%) |  |
| **Religion** | | | | |
| No religion | 20 (83.3%) | 11 (91.7%) | 9 (75 %) | 0.22^b^ |
| Buddism | 3 (12.5%) | 0 (0%) | 3 (25%) |  |
| Christianity | 1 (4.2%) | 1 (8.3%) | 0 (0%) |  |
| **Employment status** | | | | |
| Currently employed | 14 (58.3%) | 9 (75%) | 5 (41.7%) | 0.21^b^ |
| Currently not employed | 10 (41.7%) | 3 (25%) | 7 (58.3%) |  |
| **Place of living** | | | | |
| City | 16 (66.7%) | 8 (66.7%) | 8 (66.7%) | 1.0^b^ |
| Village | 8 (33.3%) | 4 (33.3%) | 4 (33.3%) |  |
| **Marital status** | | | | |
| Married | 15 (62.5%) | 8 (66.7%) | 7 (58.3%) | 1.0^b^ |
| Single/ Divorced/Separated/ Widowed | 9 (37.5%) | 4 (33.3%) | 5 (41.7%) |  |
| **Income** | | | | |
| >5 million VND ($210) | 13 (54.2%) | 7 (58.3%) | 6 (50%) | 1.0^b^ |
| 5-10 million VND | 9 (33.3%) | 4 (25%) | 5 (41.7%) |  |
| < 10 million VND | 2 (8.3%) | 1 (8.3%) | 1 (8.3%) |  |

*MCP: Music intervention combined with progressive muscle relaxation group, SD: standard deviation VND: Vietnam Dong *Significant level of p-value was set at ≤ 0.05; a= Mann-Whitney U test; b= Fisher’s exact test*

Breast cancer accounted for most of the participants (79.2%). The majority of participants were on stage 4 cancer (45.8%). The mean time from diagnosis was 20.04 months. Half of the participants were treated with docetaxel, whereas paclitaxel was treated for another 29.2%. Other chemotherapy regimens, such as gemcitabine, vinorelbine, and capecitabine, accounted for 20.8%. No significant differences were found regarding cancer stage, time from diagnosis, and chemotherapy regimen. However, there was a significant difference in cancer diagnosis between groups (p=0.037) (see detail in Table 3).

**Table 3: Baseline clinical characteristics of the participants by group assignment.**

| **Variables** | **Total (N=24)** | **Group assignment** | | **p** |
| --- | --- | --- | --- | --- |
|  |  | **MCP group**  **(n=12)** | **Control group (n=12)** |  |
| **Cancer Diagnosis** | | | | |
| Breast cancer | 19 (79.2%) | 12 (100%) | 7 (58.3%) | 0.037^b*^ |
| Gynaecological cancer | 5 (20.8%) | 0 (0%) | 5 (41.7%) |  |
| **Cancer stage** | | | | |
| Stage 1 | 3 (12.5%) | 1(8.3%) | 2 (16.7%) | 0.88^b^ |
| Stage 2 | 6 (25%) | 4 (33.3%) | 2 (16.7%) |  |
| Stage 3 | 4 (16.7%) | 2 (16.7%) | 2 (16.7%) |  |
| Stage 4 | 11(45.8%) | 5 (41.7%) | 6 (50%) |  |
| **Time from diagnosis (Months)**  **Mean (SD)[Range]** | 20.04 (28.0)  [1-108] | 10.29 (10.47)  [3-36] | 29.17 (36.72)  [1-108] | 0.47^a^ |
| **Chemotherapy Regimen** | | | | |
| Docetaxel | 12 (50%) | 8 (66.7%) | 4 (33.3%) | 0.18^b^ |
| Paclitaxel | 7 (29.2%) | 3 (25%) | 4 (33.3%) |  |
| Others | 5 (20.8%) | 1 (8.3%) | 4 (33.3%) |  |

*MCP: Music intervention combined with progressive muscle relaxation group, SD: Standard deviation *Significant level of p-value was set at ≤ 0.05; a= Mann-Withney U test; b= Fisher’s exact test*

**3.2.2. Preliminary effects of the intervention on outcome measures across time**

Table 4 compares the mean of outcomes at time points and mean changes from baseline between the intervention and control groups. It reported that no significant differences were found regarding baseline outcomes between groups. A comparison of the change in T0 and T1 between two arms revealed that MI combined with PMR significantly reduced anxiety with a medium effect size (g= 0.62, p=0.02). A trend toward a greater reduction was noted in anxiety in the intervention group at T2 with a medium effect size, yet no significant difference was observed between the two groups (g= 0.57, p=0.28). Similarly, there was a trend of decrease in depression (g=0.23 at T1 and 0.66 at T2), stress (g=0.35 at T1 and 0.01 at T2) and an improvement trend in QoL (g= 0.47 at T1 and 0.89 at T2) in the intervention group than that of the control group. However, the differences were not statistically significant between the two groups.

**Table 4. Outcome measures across time between the groups.**

| ***Outcomes*** | **Intervention**  **(n=12)** | **Control**  **(n=12)** | **Effect size** | **p-value^a^** |
| --- | --- | --- | --- | --- |
| ***Anxiety score (range: 0-42)*** | | | | |
| T0 | 9.67(4.81) | 9.83(11.07) |  | 0.29 |
| T1 | 8.73 (9.64) | 13.83 (11.68) |  | 0.2 |
| T2 | 8.22 (8.45) | 14.0 (13.02) |  | 0.34 |
| Change (T1-T0) | -1.27 (9.93) | 4.0 (6.98) | 0.62 | **0.02*** |
| Change (T2-T0) | -1.56 (5.36) | 3.45 (10.81) | 0.57 | 0.28 |
| ***Depression score (range: 0-42)*** | | | | |
| T0 | 7.17 (8.07) | 10.67 (9.99) |  | 0.38 |
| T1 | 7.45 (10.55) | 11.83 (12.52) |  | 0.33 |
| T2 | 7.11 (10.68) | 12.9 (12.27) |  | 0.1 |
| Change (T1-T0) | -1.18 (12.44) | 1.17 (7.26) | 0.23 | 0.66 |
| Change (T2-T0) | -0.67 (3.46) | 2.18 (4.93) | 0.66 | 0.16 |
| ***Stress score (range: 0-42)*** | | | | |
| T0 | 11.67 (5.71) | 15.17 (9.08) |  | 0.27 |
| T1 | 11.09 (11.36) | 17.33 (12.49) |  | 0.2 |
| T2 | 12 (8.06) | 14.9 (13.19) |  | 0.7 |
| Change (T1-T0) | -1.27 (12.2) | 2.17 (7.26) | 0.35 | 0.25 |
| Change (T2-T0) | -0.67 (6.25) | -0.55 (9.96) | 0.01 | 0.73 |
| **QOL overall (range: 0-108)** | | | | |
| T0 | 78.51 (12.25) | 76.55(17.3) |  | 0.67 |
| T1 | 77.48 (16.26) | 71.1(18.85) |  | 0.32 |
| T2 | 82.35 (15.46) | 69.28 (23.87) |  | 0.19 |
| Change (T1-T0) | 0.38 (13.89) | - 5.44 (10.83) | 0.47 | 0.22 |
| Change (T2-T0) | 4.34 (13.6) | -6.41 (10.49) | 0.89 | 0.12 |

*Data are presented as mean (standard deviation) at time points*

*Abbreviations: T0, baseline; T1, 3 weeks from baseline; T2, 6 weeks from baseline*

*# Hedges’g effect size which corresponds to the standardized mean difference of the mean changes at the underlying time point with respect to T0 between the intervention and control groups , *Significant level of p-value was set at ≤ 0.05; a=Mann- Whitney U test*

3.3. **The acceptability of the intervention**

A total of ten participants completed the semi-structured interview, and two themes were generated regarding the acceptability of the intervention. The themes were: (1) Beneficial effect on psychological and physical health and (2) Willing to keep practising in the future.

**Theme 1: Beneficial effect on psychological and physical health**

Most participants reported that they had better psychological and physical health after three-weeks of self- practice at home. Participants reported feeling comfortable, relaxed, and calm.

“I *feel more comfortable in my body, more relaxed, and my mind is more relaxed (P2)”.*

Besides, some participants reported positive effects on physical health such as reduced nausea, vomiting, pain, and insomnia.

*“During the practice, I was nauseous and uncomfortable; at that time, I just focused, and then I got better and forgot the feeling of nausea. After that, I kept focusing on the forehead area, focusing on it all, focusing on only one position. My legs were the same, my arms were the same, my breathing was the same, and I didn't think about things (P3).”*

**Theme 2: Willing to keep practising in the future**

The participants could feel the positive impacts on their health after the intervention. All the participants were willing to continue practising the intervention in the future and wanted to share the intervention with other cancer patients.

*“I think I will practice often; for example, when I finish chemotherapy, I have side effects as you said; when I focus on something, I forget the feeling. I'll feel better then. I think it's also better because when I focus on this, I forget that feeling. Or maybe, like two days ago, I told you I had pain here (pointing to her stomach). Then, when I practised those two days, I focused on listening to music and fell asleep. Normally, I have a hard time sleeping, and I can't sleep. If I can't sleep, I practice like that. Then I try to practice and see if I listen to the music, the music is also gentle, I think it's easier to sleep, I don't think so then I listen to it, and then I finish practising, I go to sleep. Every day, it's very difficult to sleep like I have pain, the day I told my sister that I didn't understand why this time I went to transmit it for some reason, but I kept hurting all the time. The pain here is like I have a stomach ache; I go to sleep so hard I can't sleep. When I'm done practising, I feel like I relax, I listen to the music, and then I fall asleep, I forget the pain (P4)”*

4. **Discussion**

Breast and gynaecological cancer patients receiving chemotherapy have a significant risk of psychological issues because of various factors such as side effects of the treatment, sexual issues, and changes in body image [35-37]. This pilot randomized controlled trial provided a novel way that combined MI with PMR to solve psychological issues commonly faced by breast and gynaecological cancer patients receiving chemotherapy. The pilot study results showed a promisingly high feasibility of the combined intervention and a potentially effective strategy for stress management in the target population.

Our study supported the feasibility of the study with high recruitment and consent rate and acceptable attrition rate (16.7%), which was similar to the previous studies with the attrition rate ranging from 4 to 25% [38-40]. However, the reasons for dropout in our study were due to objective factors such as changing hospital or changing chemotherapy schedule because of Covid-19 infection, while a large number of dropouts in the previous studies were due to discontinued intervention [38-40]. The most challenging part of a home-based intervention is how to manage and boost the participants' adherence at home. In our study, apart from the commonly used strategies such as phone calls and distributing guidelines and forms serving as a reminder to participants, we sought support from family members of the participants. Family members were assigned to remind the participants to self-practise the intervention at home. Furthermore, the high adherence rate in our study could also be attributed to the materials and Mp3 recording we have prepared. These materials were carefully assessed by an expert panel, which ensured the scientific standard and adapted cultural elements of the population, such as language, music genre, and tempo.

Our study indicated that the combined intervention positively impacted in anxiety reduction with a medium effect size in the breast and gynaecological cancer receiving chemotherapy. It differed from a previous study by Zhou, Li [41], which showed that the combined MI and PMR could reduce anxiety with a large effect size (Cohen's d= 1.74). Firstly, K. Zhou’s study assessed for only a short period- from admission to discharge (range from 11 to 21 days) after radical mastectomy, while our research evaluated longer- after three weeks from baseline. Thus, the difference in the assessment time frame might produce different results. Secondly, K. Zhou’s study delivered the intervention during hospitalization, which was not a home-based intervention; hence, all sessions were supervised by instructors. Therefore, the participants’ social condition might have contributed to producing more effective results. Other outcomes did not show any statistically significant results, yet the study indicated a trend-forward of a higher decrease in depression, stress and improving QoL in the intervention group than that of the control group with a small to large effect size. Similar to the previous study, MI combined with PMR could help to decrease depression levels in breast cancer patients after radical mastectomy with a medium effect size (Cohen's d= 0.72). Another previous study testing the effect of this combined intervention among intensive care nurses showed some positive effects on stress reduction [42].

Regarding QoL, Akmeşe & Oran determined the effect of 20 minutes of PMR accompanied by low-level background music on pregnant women for eight weeks [43]. The findings indicated that the intervention group showed significant higher QoL scores after the intervention. In our study, the non-significant results might be due to the small sample size decreasing the study's statistical power and confidence level. Despite these, the intervention was acceptable to the participants as they displayed some positive effects on their physical and psychological health. Thus a large-scale RCT is warranted to test the intervention effects on the outcomes, depression, stress and QoL.

**Limitation**

There are several limitations that need to be acknowledged. The small sample size is the first limitation. Larger sample size in further studies is warranted to evaluate the effectiveness of the intervention among women with breast and gynaecological cancer receiving chemotherapy. Secondly, this was conducted in a single centre, which is not representative of the population. In future studies, it is necessary to conduct in multiple centres. Finally, it is impossible to blind participants or interveners because of the nature of the study [44], which may be a potential risk of bias influencing the study results.

**Conclusion**

This study indicated that MI combined with PMR was feasible and acceptable to women with breast and gynaecological cancer undergoing chemotherapy. The preliminary results suggest that the combined intervention could be used to help manage anxiety, depression and stress in the target population. It is recommended that subsequent studies with larger sample sizes and multiple centres are needed.

**Supporting information**

**S1 CONSORT Checklist**

**S2 Study protocol**

**Acknowledgements**

The authors would like to acknowledge all staff in Chemotherapy Unit H5, International Cooperation & Scientific Research Unit, Hanoi Oncology hospital for their support during data collection. We would like to thank Dr K C Choi, The Chinese University of Hong Kong, for his kind assistance in the statistical support. We would like to thank Dr. Thapa Chura Bahadur for his support in language editing. We would like to express our sincere thanks to Prof. Debra Burns, School of Engineering and Technology, US; Prof. John Mondanaro, The Louis Armstrong Department of Music Therapy, US; Prof. Zehra Gok Metin Hacettepe University Faculty of Nursing, Internal Medicine Nursing Department, Turkey, Ms Hoang Thi Thanh Hue, Vietnam-France Psychology Institute, Vietnam for help with content evaluation of the intervention.

**Authors' contributions**

The idea for the review was from K T N, C WH C. The methodology was guided by C WH C, H TX H, D NS C. Delivering the intervention was performed by K T N. Q V B managed the data collection process. Data analysis was performed by K T N and H TX H. The first draft of the manuscript was written by K T N, and all authors commented and revised the original versions of the manuscripts. All authors read and approved the final manuscripts. The whole process of the review was supervised by C WH C.

**References**

1.	Karavasiloglou N, Pestoni G, Faeh D, Rohrmann S. Post-diagnostic diet quality and mortality in females with self-reported history of breast or gynecological cancers: Results from the third national health and nutrition examination survey (NHANES III). Nutrients. 2019;11(11):2558.

2.	Sung H, Ferlay J, Siegel RL, Laversanne M, Soerjomataram I, Jemal A, et al. Global cancer statistics 2020: GLOBOCAN estimates of incidence and mortality worldwide for 36 cancers in 185 countries.  CA Cancer J Clin. 2021;n/a(n/a).

3.	Bailly C, Thuru X, Quesnel B. Combined cytotoxic chemotherapy and immunotherapy of cancer: modern times. NAR Cancer. 2020;2(1):zcaa002.

4.	Bilgic S, Acaroglu R. Effects of Listening to Music on the Comfort of Chemotherapy Patients. West J Nurs Res. 2017;39(6):745-62.

5.	Velten DB, Zandonade E, de Barros Miotto MHM. Prevalence of oral manifestations in children and adolescents with cancer submitted to chemotherapy. BMC Oral Health. 2017;17(1):49.

6.	Nikbakhsh N, Moudi S, Abbasian S, Khafri S. Prevalence of depression and anxiety among cancer patients. Caspian J Intern Med. 2014;5(3):167-70.

7.	So WKW, Marsh G, Ling WM, Leung FY, Lo JCK, Yeung M, et al. Anxiety, depression and quality of life among Chinese breast cancer patients during adjuvant therapy. Eur J Oncol Nurs. 2010;14(1):17-22.

8.	Hipkins J, Whitworth M, Tarrier N, Jayson G. Social support, anxiety and depression after chemotherapy for ovarian cancer: A prospective study. Br J Health Psychol. 2004;9(4):569-81.

9.	Ballenger JC. Anxiety and depression: optimizing treatments. Prim Care Companion J Clin Psychiatry. 2000;2(3):71.

10.	Can G, Erol O, Aydiner A, Topuz E. Non-pharmacological interventions used by cancer patients during chemotherapy in Turkey. Eur J Oncol Nurs. 2011;15(2):178-84.

11.	Chirico A, Maiorano P, Indovina P, Milanese C, Giordano GG, Alivernini F, et al. Virtual reality and music therapy as distraction interventions to alleviate anxiety and improve mood states in breast cancer patients during chemotherapy. J Cell Physiol. 2020;235(6):5353-62.

12.	Lerman C, Rimer B, Blumberg B, Cristinzio S, Engstrom PF, MacElwee N, et al. Effects of coping style and relaxation on cancer chemotherapy side effects and emotional responses. Cancer Nurs. 1990;13(5):308-15.

13.	Song QH, Xu RM, Zhang QH, Ma M, Zhao XP. Relaxation training during chemotherapy for breast cancer improves mental health and lessens adverse events. Int J Clin Exp Med. 2013;6(10):979-84.

14.	Yilmaz SG, Arslan S. Effects of progressive relaxation exercises on anxiety and comfort of Turkish breast cancer patients receiving chemotherapy. Asian Pac J Cancer Prev. 2015;16(1):217-20.

15.	Jasemi M, Aazami S, Zabihi Roghaieh E. The Effects of Music Therapy on Anxiety and Depression of Cancer Patients. Indian J. Palliat. Care. 2016;22(4):455‐8.

16.	Bulfone T, Quattrin R, Zanotti R, Regattin L, Brusaferro S. Effectiveness of music therapy for anxiety reduction in women with breast cancer in chemotherapy treatment. Holist Nurs Pract. 2009;23(4):238-42.

17.	Sin WM, Chow KM. Effect of music therapy on postoperative pain management in gynecological patients: a literature review. Pain Manag Nurs. 2015;16(6):978-87.

18.	Lima TU, Moura ECR, Oliveira CMBd, Leal RJDC, Nogueira Neto J, Pereira EC, et al. Impact of a Music Intervention on Quality of Life in Breast Cancer Patients Undergoing Chemotherapy: A Randomized Clinical Trial. Integr Cancer Ther. 2020;19:1534735420938430.

19.	Nguyen KT, Xiao J, Chan DNS, Zhang M, Chan CWH. Effects of music intervention on anxiety, depression, and quality of life of cancer patients receiving chemotherapy: a systematic review and meta-analysis. Support Care Cancer. 2022;30(7):5615-26.

20.	Chen SC, Yeh ML, Chang HJ, Lin MF. Music, heart rate variability, and symptom clusters: a comparative study. Support Care Cancer. 2020;28(1):351-60.

21.	Zhou KN, Li XM, Li J, Liu M, Dang SN, Wang DL, et al. A clinical randomized controlled trial of music therapy and progressive muscle relaxation training in female breast cancer patients after radical mastectomy: Results on depression, anxiety and length of hospital stay. Eur J Oncol Nurs. 2015;19(1):54-9.

22.	Robb SL. Music Assisted Progressive Muscle Relaxation, Progressive Muscle Relaxation, Music Listening, and Silence: A Comparison of Relaxation Techniques. J Music Ther. 2000;37(1):2-21.

23.	Kibler VE, Rider MS. Effects of progressive muscle relaxation and music on stress as measured by finger temperature response. J Clin Psychol. 1983;39(2):213-5.

24.	Liao J, Wu Y, Zhao Y, Zhao Y-C, Zhang X, Zhao N, et al. Progressive Muscle Relaxation Combined with Chinese Medicine Five-Element Music on Depression for Cancer Patients: A Randomized Controlled Trial. Chin J Integr Med. 2018;24(5):343-7.

25.	Mor V, Laliberte L, Morris JN, Wiemann M. The Karnofsky performance status scale: An examination of its reliability and validity in a research setting. Cancer. 1984;53(9):2002-7.

26.	Julious SA. Sample size of 12 per group rule of thumb for a pilot study. Pharm Stat. 2005;4(4):287-91.

27.	Clark L, Dean A, Mitchell A, Torgerson DJ. Envelope use and reporting in randomised controlled trials: A guide for researchers. Research Methods in Medicine & Health Sciences. 2020:2632084320957204.

28.	Efird J. Blocked randomization with randomly selected block sizes. Int J Environ Res Public Health. 2011;8(1):15-20.

29.	Nguyen KT, Vu THN, Tran TTM, Chan WHC. A qualitative study on sterss, coping, and percieved music intervention among women with cancer recieving chemotherapy during covid-19 pandemic in Vietnam the 47th Annual ONS Congress; March 2022; California, US: Oncol Nurs Forum; 2022. p. 14-5.

30.	Bro ML, Johansen C, Vuust P, Enggaard L, Himmelstrup B, Mourits-Andersen T, et al. Effects of live music during chemotherapy in lymphoma patients: a randomized, controlled, multi-center trial. Support Care Cancer. 2019;27(10):3887-96.

31.	Noh H-E. The effects of vocal vs. instrumental music on stress relief as measured through anxiety levels: Michigan State University; 2009.

32.	Lovibond S, Lovibond P. Manual for the depression anxiety stress scales. 2nd edn. Sydney: Psychology Foundation, 1995. Google Scholar. 1995:4-42.

33.	Tran TD, Tran T, Fisher J. Validation of the depression anxiety stress scales (DASS) 21 as a screening instrument for depression and anxiety in a rural community-based cohort of northern Vietnamese women. BMC Psychiatry. 2013;13(1):24.

34.	Perdices M. Null hypothesis significance testing, p-values, effects sizes and confidence intervals. Brain Impairment. 2018;19(1):70-80.

35.	Fobair P, Stewart SL, Chang S, D'Onofrio C, Banks PJ, Bloom JR. Body image and sexual problems in young women with breast cancer. Psychooncology. 2006;15(7):579-94.

36.	Sacerdoti RC, Laganà L, Koopman C. Altered sexuality and body image after gynecological cancer treatment: how can psychologists help? Prof Psychol Res Pr. 2010;41(6):533.

37.	Molassiotis A, Chan C, Yam B, Chan S. Quality of life in Chinese women with gynaecological cancers. Support Care Cancer. 2000;8(5):414-22.

38.	Charalambous A, Giannakopoulou M, Bozas E, Marcou Y, Kitsios P, Paikousis L. Guided imagery and progressive muscle relaxation as a cluster of symptoms management intervention in patients receiving chemotherapy: a randomized control trial. PLoS One. 2016;11(6):e0156911.

39.	Dikmen HA, Terzioglu F. Effects of Reflexology and Progressive Muscle Relaxation on Pain, Fatigue, and Quality of Life during Chemotherapy in Gynecologic Cancer Patients. Pain Manag Nurs. 2019;20(1):47-53.

40.	Gok Metin Z, Karadas C, Izgu N, Ozdemir L, Demirci U. Effects of progressive muscle relaxation and mindfulness meditation on fatigue, coping styles, and quality of life in early breast cancer patients: An assessor blinded, three-arm, randomized controlled trial. Eur J Oncol Nurs. 2019;42:116-25.

41.	Zhou K, Li X, Li J, Liu M, Dang S, Wang D, et al. A clinical randomized controlled trial of music therapy and progressive muscle relaxation training in female breast cancer patients after radical mastectomy: results on depression, anxiety and length of hospital stay. Eur J Oncol Nurs. 2015;19(1):54-9.

42.	Ozgundondu B, Gok Metin Z. Effects of progressive muscle relaxation combined with music on stress, fatigue, and coping styles among intensive care nurses. Intensive Crit Care Nurs. 2019;54:54-63.

43.	Akmeşe ZB, Oran NT. Effects of Progressive Muscle Relaxation Exercises Accompanied by Music on Low Back Pain and Quality of Life During Pregnancy. J Midwifery Womens Health. 2014;59(5):503‐9.

44.	Huang S, Good M, Zauszniewski JA. The effectiveness of music in relieving pain in cancer patients: A randomized controlled trial. Int J Nurs Stud. 2010;47(11):1354-62.
