# Supplementary material for: Effects of music intervention combined with progressive muscle relaxation on anxiety, depression, stress and quality of life among women with cancer receiving chemotherapy: A pilot randomized controlled trial
Source: PLoS One. 2023 Nov 3;18(11):e0293060. doi: 10.1371/journal.pone.0293060 (PMC10624313; doi:10.1371/journal.pone.0293060)
Supplement: Attachment — Submitted filename: renamed_a9330.docx [file pone.0293060.s005.docx]

# COMMENTS – Manuscript PONE-D-22-31423

Title: “Effects of Music Intervention combined with Progressive Muscle Relaxation on Anxiety, Depression, Stress and Quality of Life among Women with cancer Receiving Chemotherapy: A Pilot Randomized Controlled Trial”

**Important note: This review pertains only to ‘statistical aspects’ of the study and so ‘clinical aspects’ [like medical importance, relevance of the study, ‘clinical significance and implication(s)’ of the whole study, etc.] are to be evaluated [should be assessed] separately/independently. Further please note that any ‘statistical review’ is generally done under the assumption that (such) study specific methodological [as well as execution] issues are perfectly taken care of by the investigator(s). This review is not an exception to that and so does not cover clinical aspects {however, seldom comments are made only if those issues are intimately / scientifically related & intermingle with ‘statistical aspects’ of the study}. Agreed that ‘statistical methods’ are used as just tools here, however, they are vital part of methodology [and so should be given due importance]. I look at the manuscript in/with statistical view point, other reviewer(s) look(s) at it with different angle so that in totality the review is very comprehensive. However, there should be efforts from authors side to improve (may be by taking clues from reviewer’s comments). Therefore, please do not limit the revision only (with respect) to comments made here.**

**COMMENTS:** Though the topic is very important, as according ABSTRACT the aim of this study is stated as ‘to investigate the feasibility, acceptability and preliminary effects of music intervention combined with progressive muscle relaxation on anxiety, depression, stress, and quality of life’ is little questionable because there is no question of testing/knowing feasibility as well as acceptability of such intervention [which is well established & known in many types of cancer patients]. Do you expect that feasibility and acceptability will differ in cancer patients receiving chemotherapy? May please refer to quoted references in this manuscript (10-17) itself.

This study being ‘pilot’ in nature, sample size is not a big issue. However, [though many things are ignored (loosely looked at / evaluated)] methodological issues need to be very rigorous followed. As said in lines 370-372: “In our study, the non-significant results might be due to the small sample size decreasing the study's statistical power and confidence level” but remember that “Absence of evidence is not evidence of absence” [Altman DG, Bland JM. BMJ volume 311, 1995, p 485 (Reprinted: Australian Veterinary Journal 1996;74, 311)]. {Even when P-value is not significantly lower that is null hypothesis of no difference / no association is not rejected, (in short, result is not significant), that does not amount to evidence of absence i.e., it does not imply that there no difference / no association. It only implies that there is no (i.e., these samples do not provide) [say enough] evidence to prove (rather indicate with certain specified confidence level) the difference / association}. Therefore, conclusion(s) from any study [in which result(s) is/are not significant], should be drawn in the light of this fact.

I am not surprised to read lines like 285-287: “A trend toward a greater reduction was noted in anxiety in the intervention group at T2 with a medium effect size, yet no significant difference was observed between the two groups (g= 0.57, p=0.28)” in backdrop of the fact [other point to mind] that: a pilot trial is not to assess effectiveness (or efficacy) and therefore, no formal hypothesis testing for effectiveness (or efficacy). I do not understand/know, why you say “with a medium effect size” because in studies on such topic generally the ‘effect size’ is very small and so you are unlikely to find any difference while working with such small sample.

Supporting information [file S1] includes CONSORT Checklist, but there you are supposed to give exact page number(s) for each item (last column: Reported on page No.). Due to absence (lack) of this information, it is difficult to check the presence/mention of many important items (though most of important items are covered / described adequately). ‘Fig 1: The Consolidated Standards of Reporting Trial (CONSORT) flow diagram’ is perfectly alright.

According to lines 228-230, “Mann–Whitney U test was used to compare the mean difference of DASS and FACT-G between groups at baseline (T0) and T1, T2, respectively”. Note that since there are three time points which are not independent and so more appropriate test is Wilcoxon’s Signed Rank test. In fact, first apply ‘Kruskal-Wallis’ test [which is non-parametric one-way ANOVA] or preferably ‘Friedman’s test [which is non-parametric two-way ANOVA]. Also consider ‘multiple comparisons’ issue (adjust P-values yielded by Wilcoxon’s Signed Rank test). But because there are two independent groups (experimental Vs control) to be compared ultimately, best is to work on ‘change scores’. In short, for within-group two samples comparison (i.e., Paired Case) use Wilcoxon’s Signed-Ranked test, for comparison of Several Means within groups, use Two-way lay out: Friedman’s test, for between-group two samples comparison (i.e., Un-paired Case) use Mann–Whitney U test, and for comparison of Several Means - One way layout: Kruskal-Wallis’s test.

Further, since Hedges’ g (like Cohen’s d) is biased upwards for small samples (under 50), hope the correction suggested for small sample size is applied. In the context of ‘Table 2: Baseline demographic information of the participants by group assignment’, please read the following para pasted from one famous standard textbook on ‘Medical Research Methodology’.

To provide a description of baseline characteristics is entirely reasonable (since it is clearly important in assessing to whom the results of the trial can be applied), however, statistical comparison of baseline characteristics when random allocation/assignment is used/done [often for good/standard/leading journals these days] is not required, because even if P-value(s) turn(s) out to be significant (while comparing baseline characteristics despite random allocation), it is, by definition, a false positive as you then are supposed to be testing ‘randomization’ then, which in any single trial may not balance all baseline characteristics (particularly when sample sizes are small). Remember that ‘randomization’ is a sort of ‘insurance’ and not a guarantee scheme. Authors may please refer to following articles:

References:

1. Stuart J. Pocock, et al., ‘Subgroup analysis, covariate adjustment and baseline comparisons in clinical trial reporting: current practice and problems’, Statistics in medicine, 2002; 21:2917–2930 [Particularly page 2927]
2. Harrington D, et al., ‘New guidelines for statistical reporting in the journal’, N Engl J Med 2019;381:285-6

[Important message (indirectly/ultimately indicated) from these articles: Never do any comparison with respect to ‘baseline’ characteristics {by applying statistical significance test(s)}, when allocation is done randomly].

However, Statistical comparison [only with respect to important/indicated variables] of baseline characteristics may be performed, to find out if analysis adjustment (say stratified analyses or else) is required with respect to these variables.

As you know, ‘the Fisher’s exact test’ [which is commonly available in many software] is applicable to 2x2 tables only [for larger tables Exact Chi-square test is available – reference ‘A network algorithm for performing Fisher's exact test in r× c contingency tables’ Cyrus R Mehta, Nitin R Patel, Journal of the American Statistical Association, 1983, Volume: 78, Issue: 382, Pages 427-434 ], however, which one is used here (table-2 or even table-3) is not clear as otherwise how you applied when there are more than two categories {examples: ‘education’, ‘Religion’, ‘Income’}? If you have taken some category as reference (which must), what is criteria used to select the reference category? Mind you that this is a scientific/academic document and so all details should be clearly/correctly communicated (do not take readers’ for granted).

Except these few points, the study is alright. However, as pointed out in ‘important note’ above “This review pertains only to ‘statistical aspects’ of the study and so ‘clinical aspects’ should be assessed separately/independently [one should carefully consider/look at the clinical implications of the study as the implications of any study should be valuable, useful, and convincing to scientists of other discipline]. In my opinion, to rescue this article (which is quite possible), some amount of re-vision (re-drafting) may be needed. The respected ‘Editor’ may consider accepting if found ‘clinical implications’ (of this study) valuable. ‘Minor revision’ is recommended.
