# Supplementary material for: Effects of music intervention combined with progressive muscle relaxation on anxiety, depression, stress and quality of life among women with cancer receiving chemotherapy: A pilot randomized controlled trial
Source: PLoS One. 2023 Nov 3;18(11):e0293060. doi: 10.1371/journal.pone.0293060 (PMC10624313; doi:10.1371/journal.pone.0293060)
Supplement: Attachment — Submitted filename: reponse to Reviewers.docx [file pone.0293060.s006.docx]

**Title: Effects of Music Intervention combined with Progressive Muscle Relaxation on Anxiety, Depression, Stress and Quality of Life among Women with cancer Receiving Chemotherapy: A Pilot Randomized Controlled Trial**

Thank you very much for the reviewers' valuable comments on our manuscript, which we have now revised accordingly. Below are our responses to the reviewers' comments:

**Please note that all mentioned lines and pages below are on the revised manuscript with the track changes file.*

**Reviewer 1**

Keywords: use MeSH keywords

*Response: Thank you so much for your suggestion. We revised the keywords using MESH keywords in lines 82-83, page 3.*
**Abstract:**
1. Include a brief background of the study.

*Response: Thank you so much for your suggestion. A brief background was included in lines 47-49 page 2.*

2. Mention the study duration and study setting.

*Response: Thank you so much for your suggestion. The study duration and study setting were included in lines 53-54, page 2.*
3. Include the character of the study participants.

*Response: Thank you so much for your suggestion. The character of the study participants was included in lines 54-55, page 2.*
4. Include all the study outcome measures.

*Response: Thank you so much for your suggestion. The study outcome measures were included in lines 59-62, page 2.*
5. Mention the statistical tests performed for the study.

*Response: Statistics were mentioned in lines 64-65, page 2.*

6. The results should be presented with 95%CI (upper limit – lower limit) for all the variables.

*Response: The effect sizes have now been presented with 95% CIs.*

7. The conclusion should be drawn on the basis of the study reports, not on the assumption.

*Response: The conclusion was revised in lines 77-80, page 3.*
**Manuscript**
8. Mention the application procedure, it's merits and demerits with recent references.

*Response: Thank you so much for your suggestion. The application procedure, its merits and demerits with recent references were mentioned in lines 138-155, pages 5-6.*
9. Mention the gaps monitored by the researcher.

*Response: Thank you so much for your suggestion. The gaps were mentioned in lines 138-155, pages 5-6.*

10. Include the clinical significance of this study over clinicians, patients, and researchers after the study hypothesis.

*Response: Thank you so much for your suggestion. The clinical significance of this study was added in lines 164-166, page 6.*

11. Convenience sampling is not an apt method of sampling for RCT.

*Response: Since it is infeasible to compile a sampling frame for the target population, we believe convenience sampling would be an appropriate and practical approach.*

12. Mention the randomization and allocation of the subjects in detail.

*Response: Thank you so much for your suggestion. The randomization and allocation* *were included in lines 189-196, page 7.*

13. Mention who has included the study participants in the trial?

*Response: Thank you so much for your suggestion. The person who included the participants was mentioned in lines 197-198, page 7.*

14. Mention about the informed consent information.

*Response: Thank you so much for your comments. The informed obtained consent form from participants was mentioned in line 197, page 7.*

15. Mention the detail of the intervention as a separate appendix with figures for study replication.

*Response: Thank you so much for your suggestion. A detailed tensing instruction of 16 muscle groups in the sequence was provided in the supporting information (S2).*

16. Include the study outcome measures and its reliability and validity.

*Response: Thank you so much for your comments. The study outcome measures and its reliability and validity were described in lines 247-263, pages 9-10.*

17. Include the reference study for sample size calculation.

*Response: Thank you so much for your suggestion. The reference study for sample size calculation was cited and presented in the reference as "Julious SA. Sample size of 12 per group rule of thumb for a pilot study. Pharm Stat. 2005;4(4):287-91."*

18. The samples included in the study is not sufficient enough to generalize the reports.

*Response: We agree that the findings of our small sample pilot study may not be generalizable. We just want to provide some findings regarding the feasibility and acceptability as well as the preliminary effects of the music combined with progressive muscle relaxation intervention. We have reminded the readers to interpret our findings with cautions in our conclusion.*

19. The statistical tests used for the study was not apt to this study – please consult a biostatistician.

*Response: Thank you so much for your suggestion. We invited a statistician to be a co-author and review the manuscript and help address the statistical issues.*

20. Mention the test done for homogenous evaluation.

*Response: In view of the small sample size of the present pilot study and hence a likely underpowered study, we have now decided not to do any statistical test.*

21. The results should be presented with 95%CI (upper limit – lower limit) for all the variables.

*Response: Please see our response to comment #6 above.*

22. There is redundant information in the discussion part and not presented in a logical manner.

*Response: Thank you so much for your suggestion. The discussion part has been revised, some parts were rejected.*

23. Add more recent references explaining the mechanism of changing outcome variables through the administration of these intervention.

*Response: Thank you so much for your suggestion. The mechanism of changing outcomes was discussed in lines 450-466, page 20.*

24. The conclusion should be more concise and self-explanatory and drawn on the basis of study reports.

*Response: Thank you so much for your suggestion. The conclusion was revised in lines 492-498, pages 21-22.*

25. Avoid abbreviations in the conclusion.

*Response: Thank you so much for your suggestion. No abbreviation was present in conclusion.*

26. Add more real-time limitations faced by the researcher and the study.

*Response: Thank you so much for your suggestion. The* real-time limitations was added in the limitation section *in lines 483-485, page 21*.

**Reviewer 2**

1.Introduction: Explain the rationale of the study. Please delete information unrelated to objective so that the section is short and sweet. For example, the first page of introduction may be deleted. Kindly focus on three elements of introduction.

*Response: Thank you so much for your suggestion. Some unnecessary parts of the introduction were rejected in lines 100-110, 118-122, pages 3-4. And the introduction was revised following the guidance.*

2. The discussion section needs to be described scientifically. Kindly frame it along the following lines:
i. Main findings of the present study
ii. Comparison with other studies
iii. Implication and explanation of findings
iv. Strengths and limitations

*Response: Thank you so much for your suggestion. The discussion was revised following the above guidance in lines 396-490, pages 18-21.*

**Reviewer 3**

**Abstract:**

1. The background needs to be added

*Response: Thank you so much for your suggestion. A brief background was included in lines 47-48 page 2.*

2.Demographic profile of patients is not mentioned.

*Response: Thank you so much for your suggestion. The demographic profile of patients were included in lines 54-55, page 2.*

**Main text:**

3.Participants: Why you choose this age limit? And did you find cases at this limit? I think breast cancer in this age will be rare.

*Response: Thank you for your questions. This pilot study focused on adult breast and gynaecological cancer patients. It is very rare to see younger age people with BGC cancer. However, it is reported cases in* *adolescents, even children.*

*For example:*

*Gutierrez, J. C., Housri, N., Koniaris, L. G., Fischer, A. C., & Sola, J. E. (2008, 2008/06/15/). Malignant Breast Cancer in Children: A Review of 75 Patients. Journal of Surgical Research, 147(2), 182-188. https://doi.org/https://doi.org/10.1016/j.jss.2008.03.026*

*McDivitt RW, Stewart FW. Breast Carcinoma in Children.* JAMA. *1966;195(5):388–390. doi:10.1001/jama.1966.03100050096033*

4.Study sample: Explain why this type of sampling methods you used?

*Response: Since it is infeasible to compile a sampling frame for the target population, we believe convenience sampling would be an appropriate and practical approach.*

5.Study sample: Why this method you can use more accurate method for calculation to void biase

*Response: The preliminary effects of the intervention on the outcomes have now been estimated by bias-corrected Hedges'g effect sizes.*

**Reviewer 4:**

1.Though the topic is very important, as according ABSTRACT the aim of this study is stated as 'to investigate the feasibility, acceptability and preliminary effects of music intervention combined with progressive muscle relaxation on anxiety, depression, stress, and quality of life' is little questionable because there is no question of testing/knowing feasibility as well as acceptability of such intervention [which is well established & known in many types of cancer patients]. Do you expect that feasibility and acceptability will differ in cancer patients receiving chemotherapy? May please refer to quoted references in this manuscript (10-17) itself.

*Response: Yes, although the feasibility and acceptability of music or progressive muscle relaxation interventions have been well established in various types of cancer patients, the combination of both types of intervention has not been well documented, particularly in breast and gynaecological cancer patients undergoing chemotherapy.*

2. This study being 'pilot' in nature, sample size is not a big issue. However, [though many things are ignored (loosely looked at / evaluated)] methodological issues need to be very rigorous followed. As said in lines 370-372: "In our study, the non-significant results might be due to the small sample size decreasing the study's statistical power and confidence level" but remember that "Absence of evidence is not evidence of absence" [Altman DG, Bland JM. BMJ volume 311, 1995, p 485 (Reprinted: Australian Veterinary Journal 1996;74, 311)]. {Even when P-value is not significantly lower that is null hypothesis of no difference / no association is not rejected, (in short, result is not significant), that does not amount to evidence of absence i.e., it does not imply that there no difference / no association. It only implies that there is no (i.e., these samples do not provide) [say enough] evidence to prove (rather indicate with certain specified confidence level) the difference / association}. Therefore, conclusion(s) from any study [in which result(s) is/are not significant], should be drawn in the light of this fact.

*Response: Thank you for the comments. As the sample size of our pilot was small and likely underpowered to detect difference of clinical relevance. We have now decided not to do any statistical test and simply use bias corrected Hedges' g effect sizes to preliminarily estimate the effects of the intervention on the outcomes.*

3. I am not surprised to read lines like 285-287: "A trend toward a greater reduction was noted in anxiety in the intervention group at T2 with a medium effect size, yet no significant difference was observed between the two groups (g= 0.57, p=0.28)" in backdrop of the fact [other point to mind] that: a pilot trial is not to assess effectiveness (or efficacy) and therefore, no formal hypothesis testing for effectiveness (or efficacy). I do not understand/know, why you say "with a medium effect size" because in studies on such topic generally the 'effect size' is very small and so you are unlikely to find any difference while working with such small sample.

*Response: Thank you for the advice and comments. Given the small sample size and hence low precision of effect estimation, we agree that the previous interpretations could be somewhat overstated. We have now only provided the effect size estimates and their 95% confidence intervals and avoided making such kinds of interpretations*.

4. Supporting information [file S1] includes CONSORT Checklist, but there you are supposed to give exact page number(s) for each item (last column: Reported on page No.). Due to absence (lack) of this information, it is difficult to check the presence/mention of many important items (though most of important items are covered / described adequately). 'Fig 1: The Consolidated Standards of Reporting Trial (CONSORT) flow diagram' is perfectly alright.

*Response: We have now added the exact page numbers (fit with the manuscript file without track changes) for the CONSORT items.*

5.According to lines 228-230, "Mann–Whitney U test was used to compare the mean difference of DASS and FACT-G between groups at baseline (T0) and T1, T2, respectively". Note that since there are three time points which are not independent and so more appropriate test is Wilcoxon's Signed Rank test. In fact, first apply 'Kruskal-Wallis’ test [which is non-parametric one-way ANOVA] or preferably ‘Friedman’s test [which is non-parametric two-way ANOVA]. Also consider ‘multiple comparisons’ issue (adjust P-values yielded by Wilcoxon’s Signed Rank test). But because there are two independent groups (experimental Vs control) to be compared ultimately, best is to work on ‘change scores’. In short, for within-group two samples comparison (i.e., Paired Case) use Wilcoxon’s Signed-Ranked test, for comparison of Several Means within groups, use Two-way lay out: Friedman’s test, for between-group two samples comparison (i.e., Un-paired Case) use Mann–Whitney U test, and for comparison of Several Means - One way layout: Kruskal-Wallis’s test.

*Response: Thank you for the suggestions. Please refer to our response to comment #2 above.*

6. Further, since Hedges’ g (like Cohen’s d) is biased upwards for small samples (under 50), hope the correction suggested for small sample size is applied. In the context of ‘Table 2: Baseline demographic information of the participants by group assignment’, please read the following para pasted from one famous standard textbook on ‘Medical Research Methodology’.
To provide a description of baseline characteristics is entirely reasonable (since it is clearly important in assessing to whom the results of the trial can be applied), however, statistical comparison of baseline characteristics when random allocation/assignment is used/done [often for good/standard/leading journals these days] is not required, because even if P-value(s) turn(s) out to be significant (while comparing baseline characteristics despite random allocation), it is, by definition, a false positive as you then are supposed to be testing ‘randomization’ then, which in any single trial may not balance all baseline characteristics (particularly when sample sizes are small). Remember that ‘randomization’ is a sort of ‘insurance’ and not a guarantee scheme. Authors may please refer to following articles:
References:
1. Stuart J. Pocock, et al., ‘Subgroup analysis, covariate adjustment and baseline comparisons in clinical trial reporting: current practice and problems’, Statistics in medicine, 2002; 21:2917–2930 [Particularly page 2927]
2. Harrington D, et al., ‘New guidelines for statistical reporting in the journal’, N Engl J Med 2019;381:285-6
[Important message (indirectly/ultimately indicated) from these articles: Never do any comparison with respect to ‘baseline’ characteristics {by applying statistical significance test(s)}, when allocation is done randomly].
However, Statistical comparison [only with respect to important/indicated variables] of baseline characteristics may be performed, to find out if analysis adjustment (say stratified analyses or else) is required with respect to these variables.

*Response: Thank you for the comments and references. We agree not to do statistical comparisons between groups regarding baseline characteristics, particularly without prior knowledge about potentially important prognostic or confounding factors.*

7.As you know, ‘the Fisher’s exact test’ [which is commonly available in many software] is applicable to 2x2 tables only [for larger tables Exact Chi-square test is available – reference ‘A network algorithm for performing Fisher's exact test in r× c contingency tables’ Cyrus R Mehta, Nitin R Patel, Journal of the American Statistical Association, 1983, Volume: 78, Issue: 382, Pages 427-434 ], however, which one is used here (table-2 or even table-3) is not clear as otherwise how you applied when there are more than two categories {examples: ‘education’, ‘Religion’, ‘Income’}? If you have taken some category as reference (which must), what is criteria used to select the reference category? Mind you that this is a scientific/academic document and so all details should be clearly/correctly communicated (do not take readers’ for granted).
*Response: Thank you for the advice. We will bear in mind the suggestions for our future reports, as in view of the small sample size of the present pilot study and hence a likely underpowered study, we have now decided not to do any statistical tests on baseline comparison.*
