# Supplementary material for: Effects of music intervention combined with progressive muscle relaxation on anxiety, depression, stress and quality of life among women with cancer receiving chemotherapy: A pilot randomized controlled trial
Source: PLoS One. 2023 Nov 3;18(11):e0293060. doi: 10.1371/journal.pone.0293060 (PMC10624313; doi:10.1371/journal.pone.0293060)
Supplement: Attachment — Submitted filename: Reponse to Reviewers.docx [file pone.0293060.s007.docx]

**Title: Effects of Music Intervention combined with Progressive Muscle Relaxation on Anxiety, Depression, Stress and Quality of Life among Women with cancer Receiving Chemotherapy: A Pilot Randomized Controlled Trial**

Thank you very much for the valuable comments on our manuscript, which we have now revised accordingly. Below are our responses to comments of the editors and reviewers:

**A. Journal Requirements:**

1. Please review your reference list to ensure that it is complete and correct. If you have cited papers that have been retracted, please include the rationale for doing so in the manuscript text, or remove these references and replace them with relevant current references. Any changes to the reference list should be mentioned in the rebuttal letter that accompanies your revised manuscript. If you need to cite a retracted article, indicate the article’s retracted status in the References list and also include a citation and full reference for the retraction notice.

*Response: Thank you for your reminder. The reference list was carefully checked that it includes published date and a database's unique identifiers (PMID, PMCID, or DOI). There are no retracted papers was cited in the reference list.*

2. Please upload a copy of your trial study protocol as a supporting information file. By the study protocol, we mean the complete and detailed plan for the conduct and analysis of the trial that the ethics committee approved before the trial began. Please send this in the original language. If this is in a language other than English, please also provide a translation. Please detail any deviations from this study protocol in the Methods section of your manuscript. Your study protocol will be made available to the editors and reviewers, and will be published as supporting information with your manuscript if accepted for publication. (If you do not agree to this, we will not be able to publish your manuscript).

*Response: The study protocol has been uploaded as supporting information file labeled S3.*

**B. Reviewer’s comments:**

**1. Reviewer 1:** I have a suggestion - please change the study type to - A pilot study. It is not a RCT.

*Thank you so much for your suggestion. Although this is a pilot study, it follows a randomized controlled trial approach. We believe that the original title "A Pilot Randomized Controlled Trial" more accurately reflects the study design and provides clarity. Thus, we have decided to maintain the original title.*
